# Phytochemical Composition and Biological Activities of *Dracocephalum* Species Native to Kazakhstan: A Comprehensive Review

**DOI:** 10.3390/plants15111722

**Published:** 2026-06-02

**Authors:** Assel Sabiyeva, Gayane A. Atazhanova, Yana K. Levaya, Almagul S. Makhmutova

**Affiliations:** School of Pharmacy, Karaganda Medical University, Gogol Street 40, Karaganda 100017, Kazakhstan; sabievaa@qmu.kz (A.S.); atazhanova@qmu.kz (G.A.A.); mahmutova@qmu.kz (A.S.M.)

**Keywords:** Lamiaceae, *Dracocephalum*, compounds, terpenoids, flavonoids, biological activity

## Abstract

Medicinal plants are a valuable source of bioactive metabolites, and current research focuses particularly on increasing their productivity and studying previously little-known species. Of particular interest are representatives of the families Lamiaceae, Apiaceae, and Asteraceae, for which either no data exists or only brief information is available regarding their chemical composition and biological properties. In this context, the family Lamiaceae, one of the dominant families in the flora of Kazakhstan, deserves special attention. Within the republic, it is represented by 233 species, grouped into 45 genera. The genus *Dracocephalum* L. is a key component of the Lamiaceae, comprising more than 70 species grouped into four subgenera and eight sections. In ethnomedical systems, various species of the genus are used to treat respiratory diseases, fever, digestive disorders, liver and gallbladder diseases, cardiovascular pathologies, diabetes, as well as gynecological, urological, dermatological, and dental diseases. Phytochemical studies have confirmed that species of the genus *Dracocephalum* are a rich source of polyphenols, including flavonoids and phenolic acids. Approximately 22 species of the genus have been recorded in the flora of Kazakhstan, distributed in steppe and mountainous regions (Altai, Tien Shan, Zhetysu Alatau, Karatau). This review systematizes current data on the chemical composition and biological activity of *Dracocephalum* species, highlighting their pharmacological potential and the prospects for further study and utilization of Kazakhstan’s flora.

## 1. Introduction

Currently, there is particular interest in optimizing pharmaceutical production with the aim of reducing costs while simultaneously improving the efficacy, quality, and safety of medicinal products. The use of domestically sourced medicinal plant materials is economically advantageous and holds great promise. Medicinal and aromatic plants have been used in traditional medicine worldwide for millennia and continue to play an important role in treating diseases and providing the body with nutrients [1]. In recent years, there has been growing interest in plants that provide valuable raw materials and medicinal compounds, especially those used in traditional and local therapies. The Lamiaceae family includes an exceptional variety of medicinal species with traditional applications. Well-known genera of this family, such as *Salvia*, *Mentha*, and *Lavandula*, are well known for their therapeutic properties [2,3]. Another notable genus in this family is *Dracocephalum*, which includes medicinal and aromatic plants used primarily in traditional medicine.

The genus *Dracocephalum* L. (Lamiaceae) comprises valuable ornamental and essential oil (EO) plants and includes more than 70 species worldwide. The lack of specialized studies on the genus *Dracocephalum* in Kazakhstan, as well as the absence of a complete list of the genus’s species for the country, underscores the relevance of researching their distribution in the region, as well as their biological and ecological characteristics. The genus *Dracocephalum* L. was described by C. Linnaeus in 1753 as a result of the merger of the genera *Dracocephalum* and *Moldavica*. A total of 12 species of this genus were described and, based on 46 morphological differences in inflorescence structure, were classified into two groups: *Spicata* L. and *Verticillata* L. The foundations of the modern systematics of the Lamiaceae family were laid by Bentham in his monograph “Labiatarum genera et species” [4]. In 1848, Bentham proposed a system for the genus *Dracocephalum* L. Within the genus, he distinguished four sections: *Keimodracon* Benth., *Buguldea* Benth., *Moldavica* (Adans.) Benth., and *Ruyschiana* (Mill.) Benth.

Subsequently, the taxonomic circumscription of the genus underwent repeated revisions. Some later authors considered *Moldavica* as a separate genus because of differences in floral morphology, EO composition, and ecological adaptations, whereas others supported Linnaeus’s broader concept of *Dracocephalum* [2]. Modern taxonomic treatments and molecular phylogenetic studies generally support the inclusion of *Moldavica* within *Dracocephalum*, although discussions regarding the infrageneric classification and phylogenetic relationships of several species’ complexes remain ongoing. The phenolic profile of *Dracocephalum moldavica* shows similarities to other phenolic-rich genera of the Lamiaceae family, particularly *Salvia* and *Perilla*, which are well known for their high accumulation of rosmarinic acid and flavonoids. Similar to these genera, *D. moldavica* is characterized by the predominance of hydroxycinnamic acid derivatives, especially rosmarinic acid, caffeic acid derivatives, and flavones such as luteolin and apigenin glycosides. Rosmarinic acid is considered a chemotaxonomic marker of the Nepetoideae subfamily and is widely distributed in *Salvia*, *Perilla*, *Melissa*, and related genera. However, available studies indicate that *D. moldavica* may accumulate rosmarinic acid at comparatively high concentrations, particularly under in vitro culture conditions, highlighting its potential as an alternative industrial source of natural phenolic antioxidants and anti-inflammatory compounds.

The Swiss botanist John Isaac Briquet (J. Briquet) made a significant contribution to the systematics of the Lamiaceae. He systematized and processed data on the family for the publication by A. Engler and K. Prantl, *Die naturlichen Pflanzenfamilien*—The Natural System of Plant Families [5]. Plants of the genus *Dracocephalum* are used in traditional medicine and are valuable sources of biologically active polyphenolic compounds for the cosmetic, food, and pharmaceutical industries.

Some *Dracocephalum* species are key components of traditional herbal preparations, particularly in Tibet, China, Mongolia, India, and Pakistan [6,7,8], as well as in Iran [9]. These plants are widely used to treat conditions such as respiratory diseases, asthma, colds and fever, stomach disorders, hypertension, and heart disease [10,11,12]. *Dracocephalum* species are also used to relieve pain and treat dental diseases. In addition to their medicinal uses, some species are also used as food ingredients [13,14]. The benefits of herbal preparations made from *Dracocephalum* are primarily valued by local communities, and most lack scientifically validated evidence of therapeutic efficacy [15]. However, in recent years, an increasing amount of data has emerged regarding their metabolites and pharmacological activity [16,17].

The genus *Dracocephalum* L. comprises 22 species recorded in Kazakhstan, including taxa such as *Dracocephalum integrifolium*, *D. moldavica*, *Dracocephalum ruyschiana*, *Dracocephalumheterophyllum*, *Dracocephalum thymiflorum*, and other endemic or widely distributed species. There are several review publications on the genus and individual species, for example [15,18,19,20]. These works provide an extensive analysis of the botany, chemical composition, and biological activity of the genus and individual species. However, they do not consolidate or systematize data on the Kazakhstani species.

Unlike previous reviews of the genus *Dracocephalum*, which cover broad phytochemical and pharmacological aspects, this review focuses on species native to Kazakhstan and neighboring Central Asian regions. It compiles available data on taxonomy, distribution, essential oil composition, phenolic compounds, flavonoids, lignans, and biological activities of Kazakhstani species. It also summarizes regional chemotypic characteristics and endemic taxa. Special attention is given to species that were insufficiently covered in previous international reviews. This provides a Kazakhstan-oriented overview of phytochemistry and pharmacology (Table 1).

The literature search was conducted in international databases (PubMed, Scopus, Web of Science, Google Scholar, ScienceDirect, SpringerLink), as well as in regional flora catalogs and ethnobotanical sources, with a focus on studies devoted to the chemical composition and biological activity of *Dracocephalum*, including monographs, dissertations, and peer-reviewed articles from 1990 to 2026. The data show that *Dracocephalum* species distributed in Kazakhstan have traditionally been used in folk medicine to treat gastrointestinal disorders, inflammatory conditions, cardiovascular disorders, and as a sedative [21]. Phytochemical studies have identified a wide range of secondary metabolites in these species, including flavonoids, phenolic acids, saponins, lignans, terpenes and their derivatives, alkaloids, and EO components [22]. These compounds demonstrate antioxidant, antimicrobial, anti-inflammatory, hypolipidemic, and cytoprotective activity in vitro and in vivo in a number of models. Literature data confirm that Kazakhstani species of *Dracocephalum* possess significant pharmacological potential, which justifies further preclinical and clinical studies for the development of safe and effective phytopharmaceutical preparations based on them.

## 2. Taxonomy, Distribution and Botanical Characteristics

**Figure 1:**
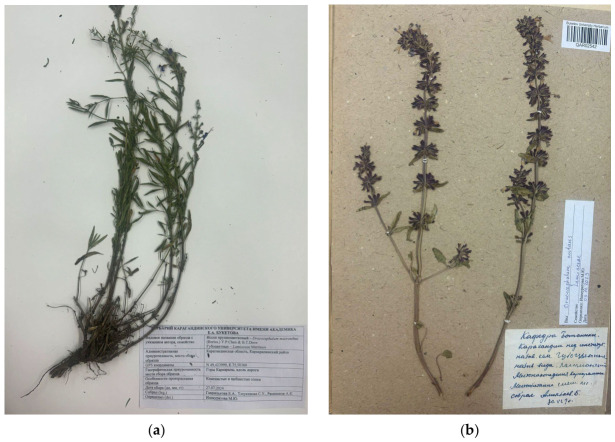
Illustrations of some herbarium specimens of Kazakhstani species: (**a**) *Dracocephalum macranthus*; (**b**) *Dracocehpalum nutans*.

*Dracocephalum* is a morphologically heterogeneous genus comprising perennial, rarely annual, herbs, sometimes woody at the base. The genus is characterized by erect or prostrate stems and flowers arranged in false whorls, forming dense spherical or loose elongated inflorescences. Taxonomically important features include the structure and pubescence of the calyx, the shape and density of inflorescences, the number of flowers per cluster, and the size and color of the corolla. The calyx is tubular or tubular-campanulate, usually 15-veined and two-lipped, while the corolla is distinctly bilabiate with a widened upper part. The genus has four stamens, with the upper pair usually longer, and smooth oblong achenes. Species adapted to high-altitude environments typically possess compact inflorescences, whereas steppe and lowland species tend to have elongated and looser ones, reflecting ecological specialization and adaptation to pollination conditions. The genus includes 38 species in the former USSR, of which 22 occur in Kazakhstan. Illustrations of some herbarium specimens of species distributed in Kazakhstan are presented in Figure 1. Characteristic features of some Kazakhstani species are presented in Table 2.

Thus, the species of the genus *Dracocephalum* found in Kazakhstan exhibit significant morphological and ecological diversity while retaining the key diagnostic features of the genus and the family Lamiaceae. They typically have erect or ascending tetrahedral stems, opposite leaves, inflorescences in the form of false whorls, and a two-lipped corolla, most often blue or purple. Most Kazakhstani representatives of the genus are perennial herbaceous plants, whereas annual forms are much rarer and are represented mainly by the species *D. moldavica* and *D. thymiflorum*. Morphological differences among species are evident in plant height, the degree of branching and stem pubescence, the shape and size of the leaf blade, the density and structure of the inflorescences, and the size of the calyx and corolla. Ecological adaptations also play a significant role: species adapted to mountainous and extreme conditions typically have more compact inflorescences, shorter shoots, and often pronounced pubescence, whereas steppe and lowland species are distinguished by taller stems and elongated inflorescences. Systematically, Kazakhstani species are distributed among several sections of the genus *Dracocephalum*, among which *Ruyschiana*, *Moldavica*, and *Dracocephalum* (*Buguldea*) are the most represented. This distribution reflects the diversity of morphological types within the genus and indicates their evolutionary and ecological differentiation under Central Asian conditions. Overall, the flora of Kazakhstan is characterized by a high species diversity of *Dracocephalum*, including both widespread and endemic or locally restricted species, which underscores the importance of further studying their systematics, morphology, and geographic distribution.

## 3. Essential Oil Composition and Chemotypic Diversity

The EOs of *Dracocephalum* are dominated by monoterpenes and their oxygenated derivatives, while sesquiterpenes occur in lower amounts (Table 3).

**Figure 2:**
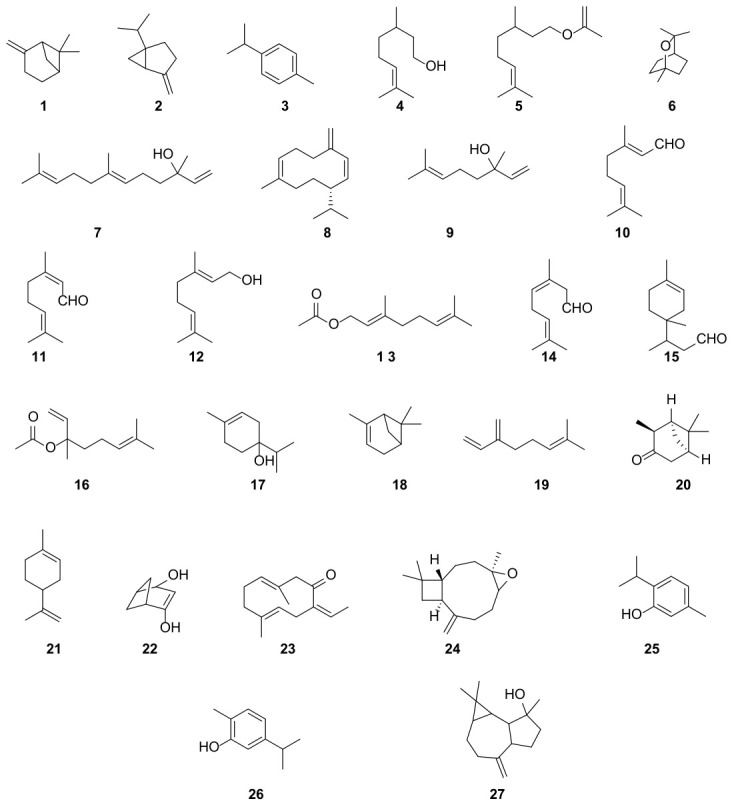
Mono- and sesquiterpenoids in essential oils identified in plants of the genus *Dracocephalum*.

The dominant components are β-pinene (**1**), geranial (**10**), neral (**11**), geraniol (**12**), α-pinene (**18**), and limonene (**21**) (Figure 2). GC–MS analysis typically identifies 30–60 compounds per sample, with their proportions varying by species, population, and growing conditions.

Most studies of EOs from the genus *Dracocephalum* employ traditional and standard methods of phytochemical analysis. Oil extraction is usually performed by hydrodistillation (Clevenger apparatus), while the identification and quantitative assessment of components are carried out using GC–MS and GC–FID with the calculation of retention indices and comparison of spectra with libraries (NIST, Wiley). These approaches constitute the methodological standard and ensure the reproducibility of results. However, it should be noted that most studies are limited to the basic analytical level, without the application of advanced methods (two-dimensional gas chromatography, chiral analysis, metabolomic profiling, and multivariate statistics for chemotype clustering). This somewhat limits the possibilities for in-depth interpretation of chemotypic variability and chemotaxonomic conclusions, especially when comparing populations from different geographic regions. The composition of the essential oil of *D. oblongifolium* was analyzed using comprehensive two-dimensional gas chromatography. It was shown that the use of a more efficient analytical technique almost doubles the number of detected components. Most of the identified compounds fit a unified biosynthetic scheme, indicating the functional activity of the terpenoid biosynthetic system in this plant [92].

The marked variability in the composition of EOs from species of the genus *Dracocephalum* is closely linked to their wide geographic distribution, ecological plasticity, and adaptation to various climatic conditions. Species of the genus are distributed throughout Eurasia—in Central Asia, China, Mongolia, Iran, Siberia, the Himalayas, and Eastern Europe—where they grow in a range of habitats, from lowland temperate-continental regions to alpine and subalpine ecosystems. Within the genus, marked geographical and ecological differentiation has been identified, reflected in the chemical composition of EOs and the formation of region-specific chemotypes, determined by altitude gradients, climate, the growing season, and extraction conditions. The most widespread is the citral chemotype (geranial + neral), characteristic, in particular, of *D. moldavica*, where the total citral content reaches 40–70% [90]. Geraniol, limonene/α-pinene, and β-caryophyllene chemotypes have also been described, occurring more frequently in wild-growing species, including *D. integrifolium* and *D. ruyschiana* [16,97]. The concentration of EO in fresh raw material varies between 0.25 and 0.98%, with its composition consisting predominantly of monoterpenes, the ratio of which changes depending on the plant’s stage of development. Environmental factors significantly influence the biosynthesis of terpenes: in continental climates, the proportion of hydrocarbon monoterpenes increases, whereas under milder conditions, the content of oxygen-containing derivatives rises. Phylogenetic studies indicate that even within a single species (e.g., *D. integrifolium* and *D. moldavica*), significant interpopulation differences are observed in the content of key components such as sabinene (**2**), 1.8-cineole (**6**), geranial (**10**), nerol (**11**), geranyl acetate (**13**), and geraniol (**12**), suggesting the influence of local environmental conditions [6]. Species from mountainous and arid regions, including *D. heterophyllum* and *D. peregrinum*, are characterized by a predominance of citronellol (**4**), linalool (**9**), and other oxygen-containing monoterpenes, whereas sesquiterpene profiles and thymol chemotypes form distinct chemical clusters [23,71]. The similarity of terpene profiles among closely related species confirms the presence of chemotaxonomic relationships within the genus and also reflects the influence of both phylogenetic relationships and environmental factors (altitude, temperature, soil conditions). Taken together, the data indicate that the chemical diversity of *Dracocephalum* EOs is shaped by the combined influence of genetic and environmental factors, which is of great importance for chemotaxonomy, raw material standardization, and the pharmacological evaluation of plant resources.

**Figure 3:**
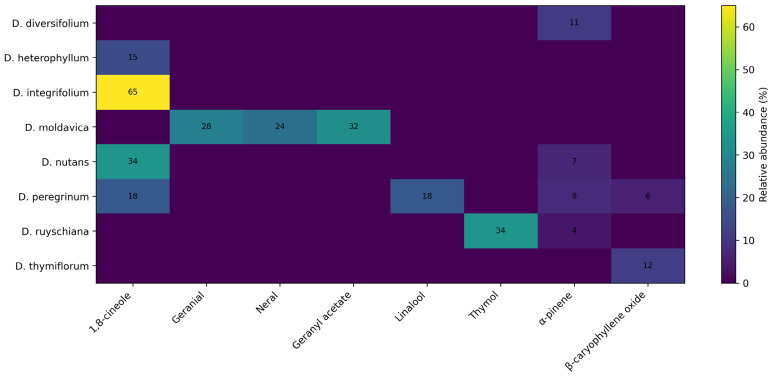
Heatmap visualization of the major essential oil constituents in different *Dracocephalum* species based on literature data. The color intensity reflects the relative abundance (%) of dominant compounds and highlights interspecific chemical variability and possible chemotypic relationships.

Heatmap analysis revealed pronounced interspecific variability in the EO composition of the genus *Dracocephalum*, enabling the identification of distinct chemotypic patterns based on dominant volatile constituents (Figure 3). Species such as *D. integrifolium* and *D. nutans* were characterized by high levels of 1.8-cineole (**6**), indicating a cineole-type chemotype. In contrast, *D. moldavica* exhibited a predominance of geranial (**10**), neral (**11**), and geranyl acetate (**13**), corresponding to a citral/geranyl acetate chemotype, while *D. ruyschiana* showed a thymol-rich profile. *D. thymiflorum* was distinguished by elevated levels of β-caryophyllene oxide, and *D. peregrinum* displayed mixed profiles containing both monoterpene hydrocarbons and oxygenated terpenoids, suggesting transitional compositional patterns. Overall, the heatmap clearly demonstrates substantial chemical diversity within the genus and supports the existence of distinct chemotypes associated with species identity and geographic origin, although this variability may also be influenced by environmental conditions and methodological differences in extraction and analysis.

Comparative analysis further confirms significant regional and ecological variability in EO composition. Species occurring in arid continental regions of Central Asia and Kazakhstan generally exhibit higher levels of monoterpene hydrocarbons, including β-pinene (**1**), α-pinene (**18**), and limonene (**21**), whereas populations from more temperate environments tend to accumulate oxygenated monoterpenes such as geranial (**10**), neral (**11**), and geraniol (**12**). Altitudinal gradients also influence chemotype formation: mountain populations typically show increased proportions of oxygenated terpenoids and more compact chemical profiles, whereas steppe and lowland populations exhibit greater variability in terpene composition. Additional environmental factors, including soil properties, temperature, humidity, and plant developmental stage, significantly affect the quantitative ratios of EO constituents.

These differences are also influenced by methodological factors, including extraction techniques (primarily hydrodistillation using a Clevenger apparatus), extraction duration, plant phenological stage, storage conditions, and analytical methods such as GC–MS and GC–FID. Variations in column type, temperature programs, identification procedures (mass spectral libraries, retention indices, and reference standards), and analytical resolution can further contribute to differences in qualitative and quantitative profiles. Together, these findings highlight the combined influence of genetic, ecological, and methodological factors on EO variability within *Dracocephalum*, with important implications for chemotaxonomy, pharmacological standardization, and practical applications.

## 4. Phenolic Compounds

Phenolic compounds constitute one of the main groups of biologically active substances in plants of the genus *Dracocephalum.* The phenolic composition of these species includes a broad spectrum of phenolic acids, flavonoids, and phenylpropanoid derivatives. These metabolites contribute substantially to the antioxidant capacity of *Dracocephalum* species and are involved in plant adaptive responses to environmental stress.

According to a recent review [20], more than 300 phenolic compounds have been identified in plants of the genus *Dracocephalum*, among which flavonoids and phenolic acids predominate. In addition, the presence of other phenolic metabolites has been noted, including anthocyanins, lignans, phenyl ethanoids, and related derivatives. The flavonoid profile is characterized by a predominance of luteolin and apigenin derivatives, whereas hydroxycinnamic acid derivatives are the most widespread among phenolic acids.

Studies of the chemical composition of various species of the genus *Dracocephalum* show that the phenolic profile may vary depending on species, growing conditions, and the developmental stage of the plants. Pigment phenolic compounds have also been identified in some species, but their distribution within the genus is limited. Collectively, these metabolites are considered major contributors to the pharmacological activity reported for *Dracocephalum* extracts [97].

In particular, the biological activity of phenolic compounds from *D. moldavica* has been experimentally confirmed in vitro. Extracts rich in rosmarinic acid exhibited strong antioxidant, iron-chelating, and antigenotoxic effects, including a significant reduction in bleomycin-induced DNA damage in human skin fibroblasts. These data experimentally confirm the biological relevance of phenolic constituents identified in *D. moldavica* [98].

In addition to the traditional therapeutic use of plant-derived products, compounds of plant origin with well-documented biological activities play an important role in the discovery of new drugs. Natural products continue to represent an important source of pharmacologically active compounds for drug discovery [89].

For *D. heterophyllum*, several studies indicate that its ethyl acetate extract exhibits the strongest biological activity. Its antioxidant properties are attributed to the presence of rosmarinic acid, acteoside, decaffeoylverbascoside, and 2-acetylplantamajoside, with IC_50_ values ranging from 19.03 to 26.63 μg/mL in the DPPH assay [98].

Zheng et al. [99] and Jiang et al. [100] report that various extract fractions from the aerial parts of *D. heterophyllum* protected against concanavalin A-induced liver injury. The treatment improved mouse survival by reducing hepatic damage, inflammatory infiltration, and the number of apoptotic cells. Additionally, the ethyl acetate extract of *D. heterophyllum* showed potential in models of non-alcoholic steatohepatitis and liver fibrosis, where it alleviated cholestasis and attenuated inflammatory responses. These effects are thought to be associated with the presence of rosmarinic acid in the extract, which may contribute to its multi-target biological activity [73].

### 4.1. Anthocyanins

Anthocyanins represent a specialized class of phenolic pigments detected in several *Dracocephalum* species. These compounds are primarily localized in the flowers and contribute to their coloration, performing the ecologically important function of attracting pollinators. In addition, anthocyanins play a role in protecting plants from various stress factors, including intense solar radiation and oxidative stress, due to their pronounced antioxidant properties.

Studies of the chemical composition of *Dracocephalum* species show that anthocyanins are not present in all species of the genus, and their content can vary significantly depending on species, growing conditions, and the stage of plant development. Compared to other groups of phenolic compounds, their distribution within the *Dracocephalum* genus is considered limited. The absence or low abundance of anthocyanins in Kazakhstani species, such as *D. moldavica*, may be associated with both ecological and genetic factors. Anthocyanin biosynthesis is known to be strongly influenced by environmental conditions, including altitude, ultraviolet radiation, temperature fluctuations, drought stress, and soil composition [47,101]. Species growing in different ecological niches may therefore exhibit distinct pigment accumulation patterns as part of their adaptive responses. In addition, interspecific differences in the regulation of flavonoid biosynthetic pathways and expression of key structural genes involved in anthocyanin formation may contribute to the observed variability among *Dracocephalum* species. However, comparative metabolomic and transcriptomic studies of Kazakhstani representatives of the genus remain limited, and further investigations are required to clarify these mechanisms. Nevertheless, the presence of anthocyanins complements the phenolic profile of these plants and may contribute to the overall antioxidant potential and biological activity of the extracts [102].

**Figure 4:**
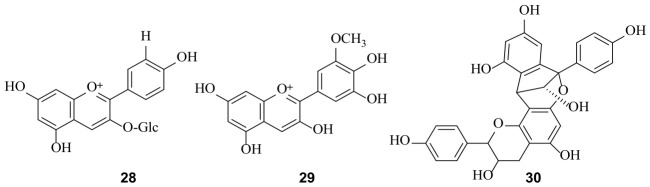
Chemical structures of anthocyanin molecules in Kazakhstani species of *Dracocephalum*, where Glc denotes a glucoside.

Anthocyanins have been detected in the aerial parts of *D. ruyschiana* by HPLC–DAD–MS analysis. These compounds were tentatively identified based on their UV–Vis and mass spectral characteristics; however, their preparative isolation and structural confirmation (e.g., by NMR spectroscopy) have not been reported. Notably, in other *Dracocephalum* species growing in Kazakhstan, anthocyanins have not been detected in the available phytochemical studies to date, suggesting possible species-specific accumulation or differences related to ecological and climatic factors; petunidin (**28**) and pelargonidin 3-O-glucoside (**29**) were detected in *D. ruyschiana* (Figure 4). Furthermore, the presence of a condensed tannin, a procyanidin A-type dimer (epicatechin-catechin dimer) (**30**), was detected in *D. ruyschiana* [102].

The anthocyanin structures shown in Figure 4 demonstrate a relatively limited but chemically significant distribution of anthocyanins among species of the genus *Dracocephalum*. The detection of petunidin (**28**) and pelargonidin (**29**) derivatives predominantly in *D. ruyschiana* indicates species-specific pathways of pigment biosynthesis and adaptation to stressful environmental conditions, particularly in high-altitude and intensely sunlit habitats. In addition to their role in pigmentation, anthocyanins may contribute to oxidative stress protection in plants. The presence of A-type procyanidin dimers further indicates the functioning of condensed tannin biosynthesis pathways, which contribute to enhancing the antioxidant and protective properties of plants.

### 4.2. Phenolic Acids

Members of the subfamily Nepetoideae Burnett are considered promising sources of bioactive compounds among plants of the family Lamiaceae Martinov due to their ability to accumulate a wide range of secondary metabolites, primarily terpenoids and polyphenolic compounds. Members of this family are characterized by a significant diversity of phenolic acids, most of which are derivatives of hydroxybenzoic and hydroxycinnamic acids.

**Figure 5:**
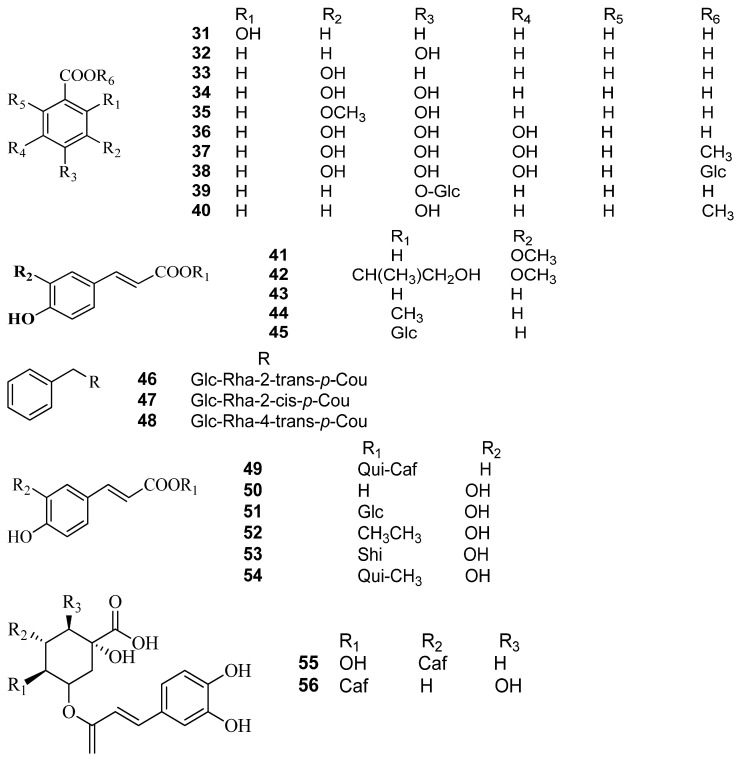
Structures of phenolic compounds (Glc denotes glucoside, Rha denotes rhamnoside, Cou denotes coumaryl, Caf denotes caffeoyl, Qui denotes quinoyl, and Shi denotes shikimoyl).

Phytochemical studies of the genus *Dracocephalum* have identified more than 300 phenolic compounds. Of particular interest are the species distributed in Kazakhstan, primarily *D. moldavica*, *D. peregrinum*, and *D. ruyschiana*, in which a wide range of phenolic acids and their derivatives has been detected (Figure 5).

Among the compounds of the hydroxybenzoic acid group in various species of *Dracocephalum*, 2-hydroxybenzoic acid (**31**), 3- and 4-hydroxybenzoic acids (**32**–**33**), 3,4-dihydroxybenzoic acid (**34**), 3-methoxy-4-hydroxybenzoic acid (**35**), as well as gallic acid (**36**) and its derivatives, including methyl gallate (**37**) and β-glucosallin (**38**). These compounds were found primarily in extracts of the aerial parts of *D. heterophyllum* [31], *D. moldavica* [86], and *D. ruyschiana* [102]. In addition, 4-(β-D-glucosyl)benzoic acid (**39**) was identified in *D. peregrinum*, while the methyl ester of p-hydroxybenzoic acid (methylparaben) (**40**) was detected in *D. moldavica* [98].

A significant portion of the phenolic compounds consists of hydroxycinnamic acid derivatives. Thus, ferulic acid (**41**) was detected in the aerial parts of *D. moldavica*, *D. peregrinum*, and *D. ruyschiana*, and its derivative—1′-methyl-2′-hydroxyethyl ferulate (**42**)—was identified in *D. peregrinum*. In addition, p-coumaric acid (**43**) and its derivatives are widespread in these species. In particular, extracts of *D. ruyschiana* contain such coumaric acid derivatives as methyl-p-coumarate (**44**), 1-O-(4-coumaroyl)-glucoside (**45**), a series of benzyl-(coumaryl)-glucosides (**46**–**48**), as well as an ester of coumaric and caffeic acids (**49**) [60,61,94,102].

Caffeic acid (**50**) is also a characteristic component of the chemical composition of *Dracocephalum* species. It has been detected in the aerial parts of *D. moldavica*, *D. peregrinum*, and *D. ruyschiana*, where it occurs both in its free form and as various derivatives (**51**–**52**) and caffeoylshikimic acid (**53**) [13,60,61,63,94,102,103].

One of the most common compounds in this group is 3-O-caffeoylquinic acid (chlorogenic acid) (**54**), which is an ester of caffeic and quinic acids. This compound is widespread in the studied species, including *D. moldavica*, *D. peregrinum*, *and D. ruyschiana*. Dicoffeoylquinic acids (**55**–**56**) have also been detected in extracts of *D. ruyschiana* [94,102,104].

Figure 5 illustrates the broad structural variability of hydroxybenzoic and hydroxycinnamic acid derivatives identified in *Dracocephalum* species. The predominance of derivatives of ferulic (**41**), coumaric (**43**), caffeic (**50**), chlorogenic (**54**) acids reflects the active phenylpropanoid metabolism characteristic of species in this genus. These metabolites likely account for much of the antioxidant and anti-inflammatory activity reported for *Dracocephalum* extracts. The occurrence of glycosylated and esterified derivatives reflects active secondary-metabolite diversification within the genus. A comparative analysis of metabolite data also confirms the chemotaxonomic significance of phenolic acids and demonstrates interspecific variability associated with ecological and geographical adaptation.

Rosmarinic acid (**57**)—an ester of caffeic acid and 3-(3,4-dihydroxyphenyl)lactic acid—occupies a special place among phenolic compounds and is widely distributed in the Lamiaceae family. This compound has been identified in *D. heterophyllum* and *D. moldavica* [13,31,104].

**Figure 6:**
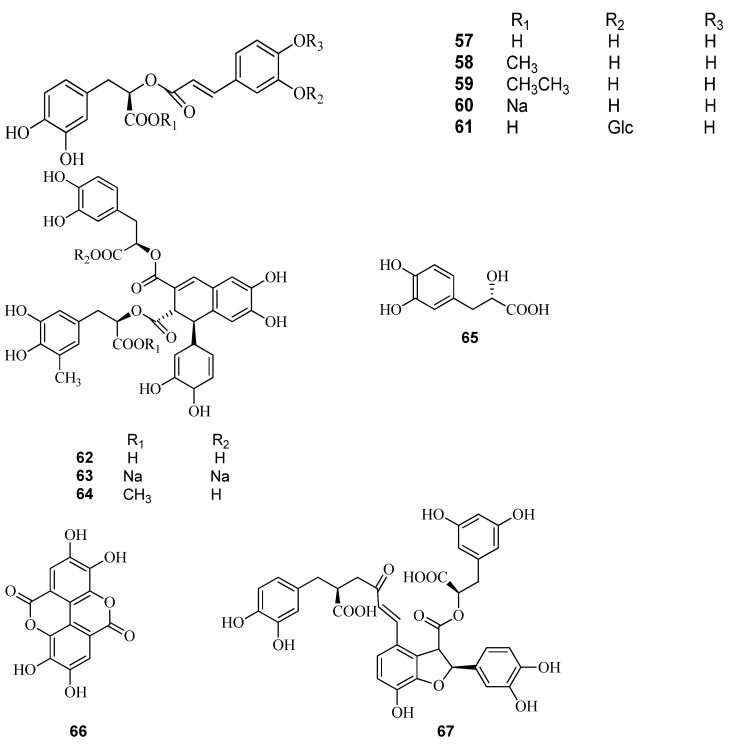
Phenolic acids found in *Dracocephalum* plants.

In addition, its derivatives (**58**–**59**) have been identified in these species, including methylrosmarinic acid (**60**) and the sodium salt of rosmarinic acid (**61**) (Figure 6). *D. moldavica* is also characterized by the presence of more complex polyphenolic compounds, such as rhabdosin (**62**) and its methyl and sodium derivatives (**63**–**64**), as well as 3-(3,4-dihydroxyphenyl)-2-hydroxypropanoic acid (danshensu) (**65**) [105].

In addition, ellagic acid—a dimer of gallic acid (**66**) [97,106]—and salvianolic acid B (**67**) [107] were detected in extracts of *D. moldavica* and *D. ruyschiana*.

Among the studied species, *D. moldavica* appears to be especially rich in phenolic acids. Caffeic (**50**), ferulic (**41**), and rosmarinic acids (**57**) have been identified in the dry extract of the herb of this species. The rosmarinic acid (**57**) content in the “Rozmatin” preparation reaches about 56.6%, while the total flavonoid content is about 27% [44]. Studies of biological activity have shown that this extract possesses a pronounced dose-dependent anti-inflammatory effect, suppressing the exudative phase of inflammation, and also exhibits significant gastroprotective activity, surpassing the effect of the reference drug omeprazole [108].

The high content of rosmarinic acid (**57**) has also been confirmed in biotechnological studies. Thus, analysis by high-performance liquid chromatography showed that in the fibrous roots of *D. moldavica*, cultivated on B5 medium under photoperiod conditions, the concentration reaches 78 mg/g dry weight, approximately ten times higher than that in the roots of plants grown under natural conditions. The obtained methanol extracts exhibited high antioxidant activity, with a pronounced ability to reduce molybdenum and neutralize DPPH radicals under the conditions [105].

Figure 6 highlights rosmarinic acid and related phenolic acids, which are among the most biologically significant metabolites of species in the genus *Dracocephalum*. The predominance of rosmarinic acid derivatives, salvianolic compounds, and caffeic acid esters confirms the accumulation of polyphenolic antioxidants characteristic of members of the Lamiaceae family. These compounds are closely associated with antioxidant, anti-inflammatory, gastroprotective, and antimicrobial activity, as confirmed by experimental studies. In particular, the exceptionally high rosmarinic acid content identified in *D. moldavica* confirms the potential of this species as a source of pharmacologically valuable phytochemical compounds. The structural diversity shown in Figure 6 also indicates the active functioning of phenolic compound biosynthetic pathways and underscores the importance of *Dracocephalum* species as sources of biologically active hydroxycinnamic acid derivatives.

Further studies using high-performance thin-layer chromatography showed that among members of the Nepetoideae subfamily, the highest rosmarinic acid content (**57**) is characteristic specifically of *D. moldavica* (24.83 ± 0.78 mg/g), highlighting the potential of this species as a promising source of phenolic metabolites [109].

Thus, an analysis of the literature indicates that members of the genus *Dracocephalum* are a valuable source of phenolic compounds, a significant proportion of which are derivatives of hydroxybenzoic and hydroxycinnamic acids. Phytochemical studies have revealed the presence of a wide range of phenolic acids, including gallic (**36**), ferulic (**41**), caffeic (**50**), chlorogenic (**54**)**,** and ellagic acids (**66**), as well as their glycoside and ester derivatives (**58**–**61**) [110,111]. Particular attention is drawn to the species distributed in Kazakhstan—*D. moldavica*, *D. peregrinum*, *and D. ruyschiana*. These plants are characterized by a significant diversity of phenolic compounds, among which derivatives of caffeic acid and complex phenolic esters play an important role. The most widely represented compound is rosmarinic acid, which has been found in significant quantities primarily in *D. moldavica* and is considered one of the main biologically active components of this species. The results of phytochemical and biotechnological studies confirm that the phenolic acids identified in species of the genus *Dracocephalum* account for their pronounced antioxidant, anti-inflammatory, and gastroprotective activity. A particularly promising source of these compounds is *D. moldavica*, characterized by a high content of rosmarinic acid and its derivatives (**58**–**61**). According to [20], rosmarinic acid derivatives have been widely identified in species of the genus *Dracocephalum* and are considered the primary compounds responsible for their biological activity. Extracts rich in rosmarinic acid demonstrated pronounced antioxidant activity in DPPH and ABTS assays, with low IC_50_ values and a high capacity for free radical scavenging. In addition, rosmarinic acid and its derivatives (**57**–**61**) exhibited significant anti-inflammatory effects by inhibiting the production of nitric oxide (NO), TNF-α, IL-6, and oxidative stress pathways in LPS-induced inflammatory models. Antimicrobial activity was also experimentally confirmed against Gram-positive bacteria and fungal strains, including *S. aureus* and *C. albicans*.

Taken together, current evidence supports the considerable phytochemical diversity of *Dracocephalum* species.

### 4.3. Flavonoids

The flavonoid profile of Kazakhstani species of the genus *Dracocephalum* is characterized by considerable diversity and generally reflects the chemotype typical of the genus. The most abundant compounds are flavones and their derivatives, suggesting that they are key components of the secondary metabolism of these plants. Along with aglycones, glycosylated forms are widespread, predominantly with sugar attachment at the C-7 position, as well as various acylated derivatives. The high degree of glycosylation accounts for the good solubility and bioavailability of these compounds, which is important from both physiological and pharmacological perspectives. This profile has been most thoroughly studied in *D. moldavica*, a species widely distributed and cultivated in Kazakhstan, where a rich spectrum of flavones and their glycosides has been identified.

In addition to flavones, flavonols are also present in the composition, represented by both free forms and various glycosides. These compounds are generally found in smaller quantities but make a significant contribution to the overall polyphenolic profile. In some cases, flavan-3-ols and their derivatives are also detected, indicating a broader spectrum of biosynthetic pathways within the genus. The presence of such compounds has also been confirmed for other Kazakhstani species, in particular *D. nutans*, which grows in the mountainous regions of Eastern Kazakhstan, where the above-ground parts have been found to contain flavonoids and other phenolic compounds. Similar patterns are observed for *D. ruyschiana* and *D. peregrinum*, although the extent of their phytochemical characterization remains limited.

A significant feature of the flavonoid composition is the presence of methoxylated derivatives, which are considered a characteristic feature of the genus. The degree of methoxylation may vary among different species and populations, reflecting the plants’ adaptive mechanisms to environmental conditions. Overall, the flavonoid profile is highly variable but retains common structural features, allowing its use in chemotaxonomic studies. For Kazakhstani representatives of the genus, including less-studied species such as *D. thymiflorum* and *D. integrifolium*, a similar set of compounds is assumed to be present, by analogy with closely related taxa.

Thus, the flavonoids of Kazakhstani species of the genus *Dracocephalum* are represented by a complex of compounds consisting primarily of flavones, as well as flavonols and other phenolic components, with a predominance of glycosylated and methoxylated forms. Their presence has been reliably established for the most studied species, primarily *D. moldavica* and *D. nutans*, and has also been noted for several other representatives of the flora of Kazakhstan. Species differences are manifested primarily in the quantitative ratio of individual groups of compounds and the presence of specific derivatives, which is due to both genetic characteristics and the influence of environmental factors.

#### 4.3.1. Flavones

**Figure 7:**
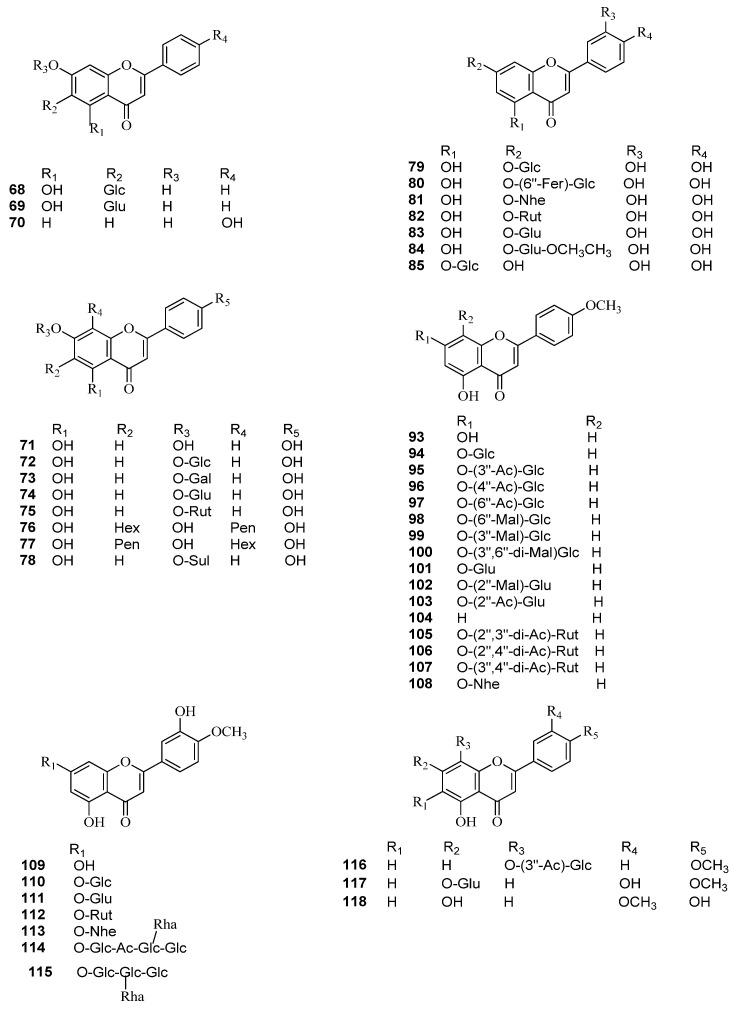
Chemical structures of flavone molecules found in *Dracocephalum* plants (Ac—acetyl, Glc—glucoside, Glu—glucuronic, Gal—galactoside, Rut—rutoside, Pen—pentoside, Hex—hexoside, Sul—sulfate, Mal—malonyl, Nhe—neohesperidoside).

Flavonoids, particularly flavone derivatives, are one of the most common groups of polyphenolic compounds found in species of the genus *Dracocephalum*. These compounds are characterized by significant structural diversity and are present as aglycones as well as numerous glycosidic, acylated, and methoxylated derivatives (Figure 7).

Among the simple flavones, various dihydroxyflavones have been identified in species of this genus. For example, chrysin (**68**) and its glucuronide (**69**) [97], whereas 7,4′-dihydroxyflavone (**70**) was found in *D. moldavica* [110].

One of the most common flavones in plants of the genus *Dracocephalum* is apigenin (5,7,4′-trihydroxyflavone) (**71**) and its derivatives. In particular, apigenin-7-O-β-D-glucoside (**72**) has been detected in the aerial parts of *D. moldavica* and *D. ruyschiana*. In addition, apigenin-7-O-galactoside (**73**) has been identified in *D. moldavica*, while apigenin-7-O-β-D-glucuronide (**74**) has been detected in *D. moldavica* and *D. ruyschiana* [44,97].

Other glycoside forms of apigenin are also characteristic of species of the genus *Dracocephalum*. Thus, apigenin-7-O-rutinoside (iso-orifolin) (**75**) has been detected in *D. heterophyllum* [31], and various C-glycosides and diglycosides of apigenin have been isolated from the aerial parts of *D. ruyschiana* (**76**–**77**). In addition, apigenin-7-sulfate (**78**) has been identified in this species [97].

A significant group of flavonoids in the genus *Dracocephalum* consists of luteolin derivatives. The most common compound in this group is luteolin-7-O-β-D-glucoside (cynaroside) (**79**), found in *D. moldavica* and *D. peregrinum* [44,97]. In *D. peregrinum*, a ferulyl derivative of luteolin-7-O-glucoside (**80**) has also been identified [94].

In addition, various glycoside forms of luteolin have been found in plants, including luteolin-7-O-neohesperidoside (**81**) in *D. moldavica*, luteolin-7-O-rutinose (**82**) in *D. heterophyllum* and *D. peregrinum*, as well as luteolin-7-O-β-D-glucuronide (**83**) and its ethyl ester (**84**), identified in *D. moldavica* and *D. ruyschiana* [97,112]. In *D. peregrinum*, plantaginine (a 7-O-glucoside of 5,6,7,4′-tetrahydroxyflavone) (**85**) has also been identified [20].

An important group of flavones in the genus *Dracocephalum* consists of methoxylated apigenin derivatives, among which the dominant compound is acacetin (5,7-dihydroxy-4′-methoxyflavone) (**86**) and its numerous derivatives (**87**–**94**). Acacetin (**86**) has been detected in *D. moldavica*, *D. peregrinum*, and *D. ruyschiana*. The most common compound in this group is acacetin-7-O-glucoside (tilianin) (87), which is widely found in species of the genus [23,97,111,112].

In addition to simple glycosides, acetylated and malonylated derivatives of tilianin (**88**–**89**) [44,113] have been identified in *D. moldavica*, as well as acacetin-7-O-β-D-glucuronide. In species of *D. heterophyllum*, *D. peregrinum*, more complex derivatives have been identified, including acacetin-7-O-rutinose (linarin) (**88**), as well as its acetylated forms (**89**–**91**), found in *D. peregrinum*; in *D. moldavica*, acacetin-7-O-neohesperidoside (**108**) [23,31,44].

Among other methoxylated flavones of the genus *Dracocephalum*, diosmetin (**109**) and its glycoside derivatives (**110**–**115**) are noteworthy. This compound has been identified in *D. heterophyllum*, *D. moldavica*, and *D. peregrinum*. Diosmetin-7-O-glucoside (**110**), diosmetin-7-O-glucuronide (**111**), as well as various diglycosides and polyglycosides of diosmetin (**112**–**115**) [15,23,31,44,97,102,112].

Diosmetin (**109**) is a biologically active natural flavonoid—the methylated aglycone of diosmin—possessing pronounced antioxidant, anti-inflammatory, and venotonic properties.

In addition to the compounds listed above, numerous polyoxygenated and polymethoxylated flavones have been identified in plants of the genus *Dracocephalum* (**116**–**125**). Other monomethoxylated flavones found in species of the genus *Dracocephalum* include dracocephaloside A—the 7-O-(3″-acetyl)glucoside of 5,8-dihydroxy-4′-methoxyflavone (**116**) and geraniol-7-O-β-D-glucuronide—the glucuronide of 5,3′-dihydroxy-4′-methoxyflavone (**117**), isolated from *D. moldavica* [110]. Chrysoeriol (5,7,4′-trihydroxy-3′-methoxyflavone) (**118**) was also detected in this species [110,114]. Dimethoxylated flavones identified predominantly in *D. moldavica* include 5,4′-dihydroxy-6,7-dimethoxyflavone (cirsimaritin) (**119**) and its 4′-O-β-D-glucoside (**120**), as well as the 4′-O-glucoside of 5,3′,4′-trihydroxy-6,7-dimethoxyflavone (**113**). In addition, 5,7,4′-trihydroxy-3′,5′-dimethoxyflavone (**114**), 5,3′,4′-trihydroxy-6,7-dimethoxyflavone (**115**), 5,8,4′-trihydroxy-6,7-dimethoxyflavone (isothymusin) (**116**), and 5,7-dihydroxy-6,4′-trimethoxyflavone (pectolaringenin) (**117**) [44,103]. These compounds have been identified primarily in the aerial parts of *D. moldavica* and *D. peregrinum*.

**Figure 8:**
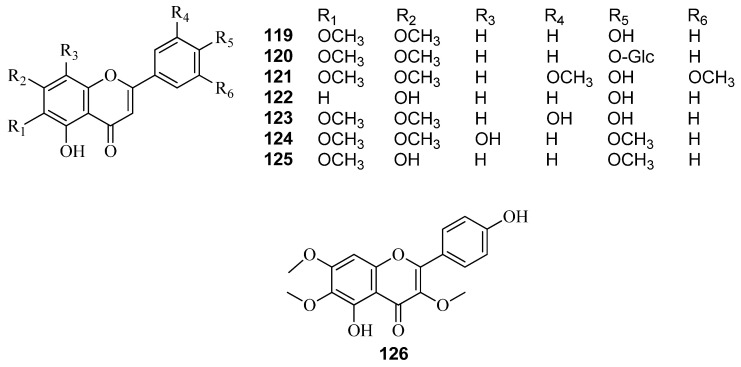
Chemical structures of flavone molecules found in *Dracocephalum* plants.

In addition, other flavonoids have been isolated from various species of the genus *Dracocephalum* (**119**–**126**) (Figure 8). Pendulethine (**126**)—a polymethoxylated flavone—was isolated from *D. komarovii* and exhibits a pronounced relaxant effect [115].

Previously, diosmetin (**109**) and cirsilineol (**117**), tilianin (**87**), luteolin-7-O-β-D-glucuronide (**83**), and apigenin-7-O-β-D-glucuronide (**74**) [41] were isolated and identified from the aerial parts of *D. komarovii* Lipsky collected in the Tashkent region (Uzbekistan).

Thus, an analysis of the literature shows that members of the genus *Dracocephalum* are characterized by a significant diversity of flavonoid compounds, among which flavone derivatives predominate. The main representatives of this group are apigenin, luteolin, acacetin, and diosmetin, as well as their numerous glycosides, acyl, and methoxylated derivatives. Particularly rich sources of flavonoids are the species *D. moldavica*, *D. peregrinum*, and *D. ruyschiana*, which are characterized by the presence of large amounts of apigenin and luteolin glycosides, as well as methoxylated flavones such as acacetin and diosmetin. The data obtained indicate the significant phytochemical potential of species of the genus *Dracocephalum*. The presence of a broad spectrum of flavonoids accounts for their pronounced antioxidant, anti-inflammatory, vasodilatory, and other pharmacological properties, confirming the promise of further research on these plants as sources of biologically active compounds and pharmacologically valuable natural substances.

#### 4.3.2. Flavonols and Their Glycosides

**Figure 9:**
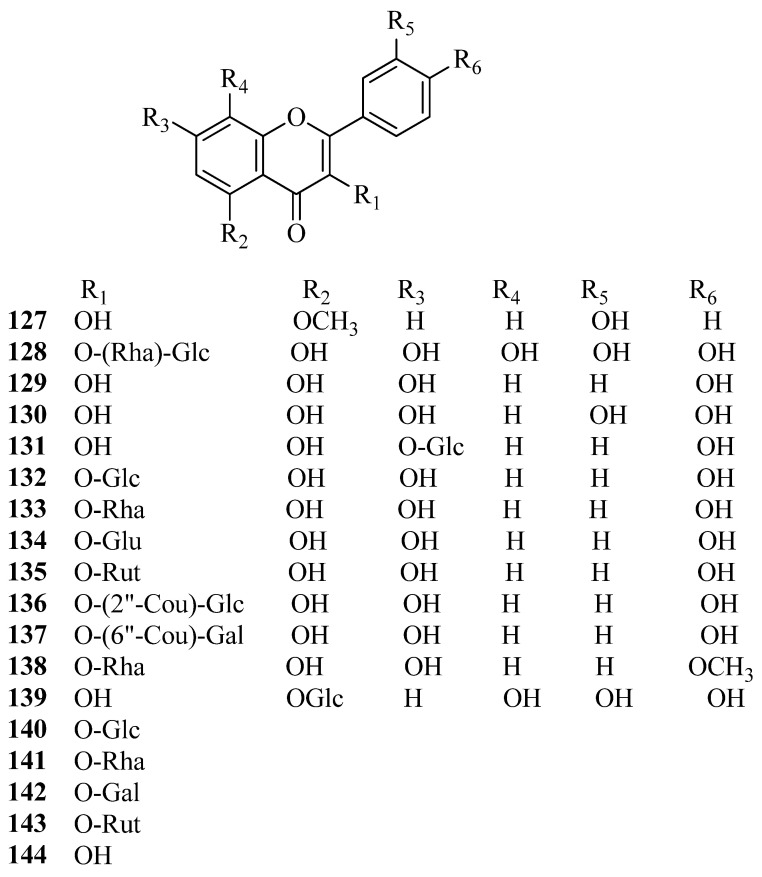
Chemical structures of flavonol molecules found in *Dracocephalum* plants (Cou—coumaryl, Glc—glucoside, Glu—glucuronic acid, Gal—galactoside, Rut—rutoside, and Rha—rhamnoside).

In addition to flavones, flavonol compounds have been identified in species of the genus *Dracocephalum*, which also make a significant contribution to the overall polyphenolic composition of plants in this genus. These compounds are present as both aglycones and numerous glycoside derivatives (Figure 9).

Among the simple flavonols in plants of the genus, 3-hydroxyflavone (**127**) was detected and identified in *D. moldavica* [116]. In addition, gossypetin (**128**)—a pentahydroxylated flavonol present as a glycoside—was identified in the aerial parts of *D. heterophyllum* [15].

The most common flavonol aglycones in the genus *Dracocephalum* are kaempferol (3,5,7,4′-tetrahydroxyflavone) (**129**) and quercetin (3,5,7,3′,4′-pentahydroxyflavone) (**130**). These compounds have been detected in the aerial parts of several species of the genus, including *D. moldavica*, *D. peregrinum*, and *D. ruyschiana*, indicating the widespread distribution of these flavonols within the genus.

Along with aglycones, a wide variety of kaempferol glycosides has been identified in *Dracocephalum* species. Among them, the most common are compounds substituted at the C-3 and C-7 positions, such as kaempferol-7-O-glucoside (**131**), astragalin (kaempferol-3-O-glucoside) (**132**), kaempferol-3-O-rhamnoside (**133**), kaempferol-3-O-glucuronide (**134**), and kaempferol-3-O-rutinose (**135**). These compounds have been identified in various species of the genus, including *D. moldavica*, *D. peregrinum*, and *D. ruyschiana* [13,15,23,31,97].

In addition, more complex kaempferol derivatives have been identified in species of the genus. For example, compounds such as 2″-coumaroylglucoside of astragalin (**136**) and kaempferol-3-O-β-D-(6″-O-p-coumaroyl)-galactoside (**137**) have been found in *D. moldavica*. In *D. peregrinum*, 3-O-rhamnoside of kaempferide (**138**)—a methoxylated derivative of kaempferol—was identified [15,23].

Quercetin glycosides are also widely represented in species of the genus *Dracocephalum.* In particular, quercetin-7-O-β-D-glucoside (**139**), quercetin-3-O-β-D-glucoside (**140**), quercitrin (quercetin-3-O-rhamnoside) (**141**), quercetin-3-O-galactoside (**142**), rutin (quercetin-3-O-rutinose) (**143**), and quercetin-3-O-β-D-glucuronide (**144**). These compounds have been identified in *D. moldavica*, *D. peregrinum*, and *D. ruyschiana*. In addition, a methoxylated derivative of quercetin—isorhamnetin (**137**) was identified in *D. moldavica* [15,23,44,86,102].

Further studies of the flavonoid composition of species in the genus *Dracocephalum* were conducted using *D. komarovii*. Several flavonoid glycosides were isolated from an ethanol extract of the aerial parts of this plant using column chromatography on silica gel, followed by purification on Sephadex LH-20. As a result, apigenin-7-O-glucoside (**72**), luteolin-7-O-glucoside (**80**), acacetin-7-O-glucoside (**88**), luteolin-7-O-glucuronide (**83**), and acacetin-7-O-[α-L-rhamnopyranosyl-(1→6)]-β-D-glucopyranoside (**89**). The structures of these compounds were determined based on data from ^1^H-NMR, ^13^C-NMR, and two-dimensional NMR experiments [41].

Thus, an analysis of the literature indicates that members of the genus *Dracocephalum* contain a variety of flavonol compounds, present as both aglycones and numerous glycoside derivatives. The main flavonols of this genus are kaempferol and quercetin, as well as their various glycoside forms, including astragalin, quercitrin, and rutin. A particularly rich composition of flavonol compounds is characteristic of the species *D. moldavica*, *D. peregrinum*, *and D. ruyschiana*, in which numerous derivatives of kaempferol and quercetin, as well as their acylated and methoxylated forms, have been identified. The presence of a wide spectrum of flavonol compounds confirms the significant phytochemical potential of species belonging to the genus *Dracocephalum.* These compounds are known for their antioxidant, anti-inflammatory, and protective biological properties, making plants of this genus promising sources of bioactive substances and subjects for further pharmacognostic and phytochemical research.

#### 4.3.3. Other Flavonoid Derivatives

**Figure 10:**
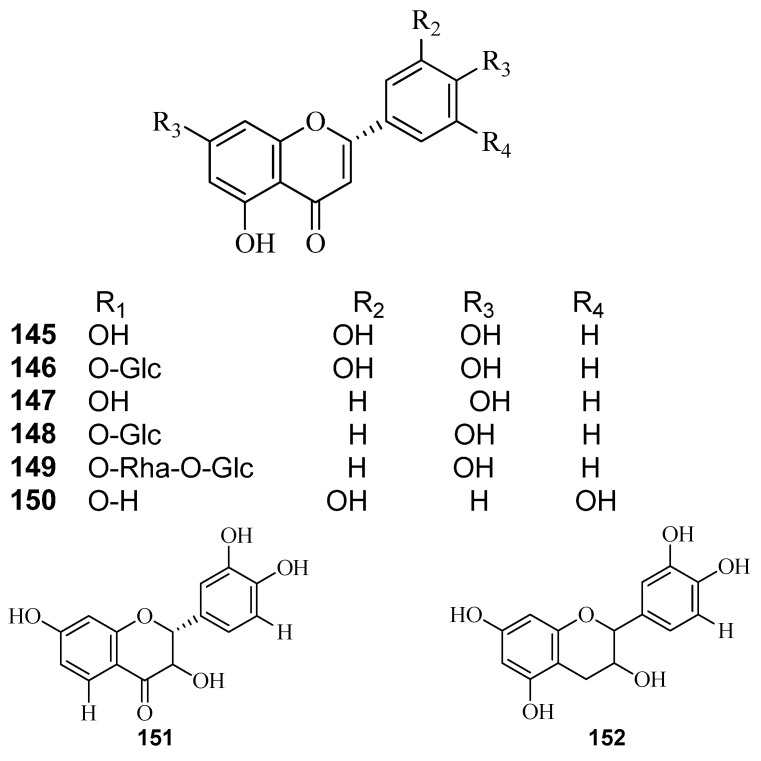
Chemical structures of flavonoid molecules found in *Dracocephalum* plants (Glc—glucoside and Rha—rhamnoside).

In addition to flavones and flavonols, representatives of other flavonoid subclasses have been identified in plants of the genus *Dracocephalum*, including flavanones, flavanonols, and flavan-3-ols (Figure 10). These compounds also contribute significantly to the biological activity of plants in this genus.

Among the flavanones, eriodictiol (**145**) has been identified in species of this genus; it was found in *D. peregrinum*, while its glycoside form (**146**) was identified in *D. ruyschiana* [20].

Another important member of this group is naringenin (5,7,4′-trihydroxyflavan-4-one) (**147**). This compound was found in *D. moldavica*, and its glycoside derivative—prunine (naringenin-7-O-glucoside) (**148**)—was identified in *D. ruyschiana* [97,107].

Further studies of the chemical composition of species in the genus have led to the identification of new flavanone compounds. Thus, (2S)-5,7,3′,5′-tetrahydroxyflavanone-7-O-β-D-glucopyranoside (**149**) and the corresponding aglycone—5,7,3′,5′-tetrahydroxyflavanone (**150**). These compounds belong to the group of flavonoid glycosides containing four hydroxyl groups and a glucose residue attached at the C-7 position [117].

The group of flavonones also includes fustin (3,7,3′,4′-tetrahydroxyflavan-4-one) (**151**), a representative of the flavanones with a tetrahydroxylated structure and belonging to the dihydroflavonol derivatives, which was detected in the aerial parts of *D. ruyschiana* [97].

In addition to flavanones, flavan-3-ols have been identified in species of the genus *Dracocephalum*. In particular, catechin (**152**) was detected in *D. ruyschiana* and *D. moldavica*. Catechin (**152**) is a flavan-3-ol belonging to the group of polyhydroxylated flavonoid derivatives and characterized by the presence of hydroxyl groups at the C-3, C-5, C-7, C-3′, and C-4′ positions. This compound belongs to the group of flavan-3-ols and is known for its pronounced antioxidant properties [20,97].

Thus, members of the genus *Dracocephalum* are characterized by the presence of various subclasses of flavonoids, including flavanones, flavanonols, and flavan-3-ols, which contribute to the overall diversity of phenolic compounds in these plants.

### 4.4. Lignans

Lignans constitute an important group of phenolic compounds found in species of the genus *Dracocephalum*. Lignans in *Dracocephalum* plants are present as phenolic dimers formed by the oxidative coupling of phenylpropanoid precursors and are characterized by moderate structural diversity and limited species distribution. These compounds are typically accumulated in small or moderate amounts and may contribute to the antioxidant and anti-inflammatory activity of plants. Their content and composition vary depending on species, plant organ, and growing conditions, allowing lignans to be considered as auxiliary components of the chemical profile of *Dracocephalum* and promising targets for further phytochemical studies. They are most widely represented in *D. moldavica*, where they can be considered characteristic components of this species’ chemical composition.

**Figure 11:**
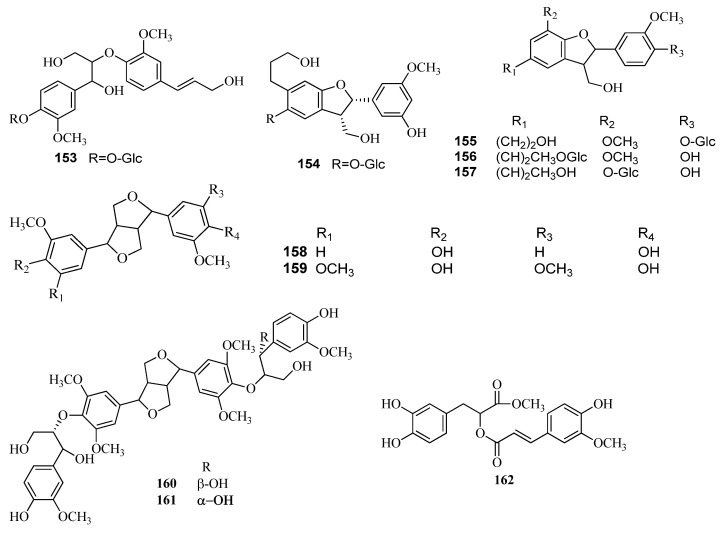
Chemical structures of lignan molecules found in *Dracocephalum* plants (Glc—glucoside).

A significant number of lignans and their derivatives have been isolated from the aerial parts of *D. moldavica* (Figure 11). Among them, alaschanisozide A (**153**), akequintoside A (**154**), various glycoside derivatives of dehydrodiconiferyl alcohol (**155**–**157**), including (7,8)-dihydrodehydrodiconiferyl alcohol-4-O-β-D-glucoside (**155**), (7,8)-dehydro-diconiferyl alcohol-9′-O-β-D-glucoside (**156**), and (7,8)-3′-demethyl-dehydro-diconiferyl alcohol-3′-O-β-D-glucoside (**157**).

In addition, well-known lignans—(+)-pinoresinol (**158**) and syringaresinol (**159**)—were identified in this species, as well as more complex compounds, including hediotizoles A and B (**160**–**161**), oresbiusin B (**162**), and some genus-specific compounds—dracomolfin A–E (**163**–**167**). Several benzofuran derivatives of phenylpropanoid origin have also been identified in *D. moldavica*. Further studies have revealed the presence of other lignans in this species, such as (+)-piperitol (**168**) and 9α-hydroxysesamin (**169**).

**Figure 12:**
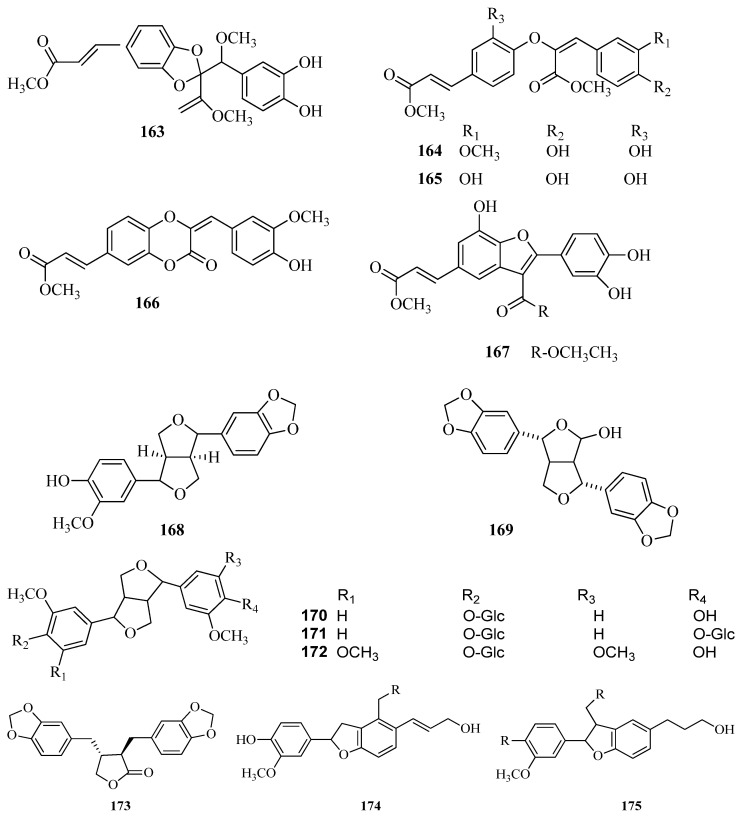
Chemical structures of lignan molecules (Glc—glucoside).

In addition to *D. moldavica*, lignans have also been identified in other species of the genus *Dracocephalum*. Specifically, the mono- and diglucosides of pinoresinol (**170**–**171**), as well as the glycoside syringaresinol (**172**), have been identified in *D. heterophyllum*, *D. moldavica* (Figure 12). The lignan quinocin (**173**) was detected in *D. ruyschiana*. Furthermore, less common lignan compounds, such as dehydropin-9-β-glucoside (**174**) and 2,3-dihydro-7-hydroxy-2-(4′-hydroxy-3′-methoxyphenyl)-3-hydroxymethyl-5-benzofuranpropanol-4′-O-β-D-glucoside (**175**) [19,20,118,119].

Thus, members of the genus *Dracocephalum* are characterized by the presence of various types of lignans, including pinoresinol, syringaresinol, and benzofuran derivatives, many of which are present as glycosides.

### 4.5. Other Classes of Secondary Metabolites

**Figure 13:**
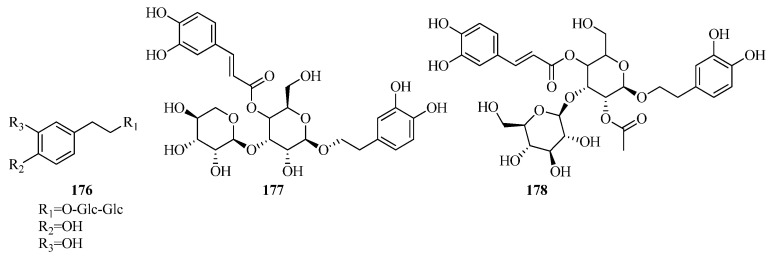
Chemical structures of phenylethanoid molecules.

In addition to phenolic acids, flavonoids, and lignans, a wide variety of other secondary metabolites has been identified in species of the genus *Dracocephalum*, including phenylethanoids, phenylpropanoids, alkaloids, stilbenes, coumarins, cyanogenic glycosides, and other phenolic compounds (Figure 13).

Phenylethanoid compounds constitute an important group of secondary metabolites in plants of the genus *Dracocephalum* and are typically represented by polar glycosylated derivatives of aromatic alcohols. They are characterized by significant structural diversity, due to differences in the degree of hydroxylation, methoxylation, and the composition of the carbohydrate moieties. These compounds play a significant role in the biological activity of *Dracocephalum* species, exhibiting antioxidant, anti-inflammatory, and antimicrobial properties. Their content and qualitative composition may vary depending on the species, plant organ, and growing conditions, which allows phenylethanoids to be considered important components of the genus’s chemical profile and promising markers for phytochemical and pharmacognostic studies. Among the phenylethanoid compounds in representatives of the genus, ferruginose B (**176**), verbascoside (acteoside) (**177**), and 2′-O-acetylplantamayoside (**178**) were identified, isolated from the aerial parts of *D. heterophyllum*. These compounds belong to the group of phenylethanoid glycosides and are known for their antioxidant and anti-inflammatory activity [120].

**Figure 14:**
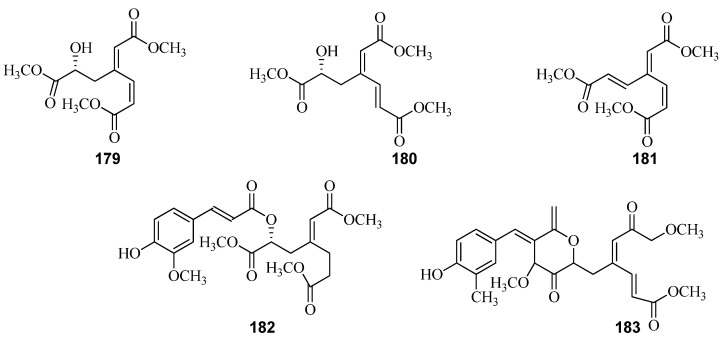
Chemical structures of phenylpropanoid molecules.

Of particular interest are unusual phenylpropanoid compounds (Figure 14). For example, five 3,4-seco-phenylpropanoids with a unique structure characterized by a cleavage of the aromatic ring were isolated from the aerial parts of *D. moldavica*. These compounds were named dracomolfesins A–E (**180**–**184**) [121].

**Figure 15:**
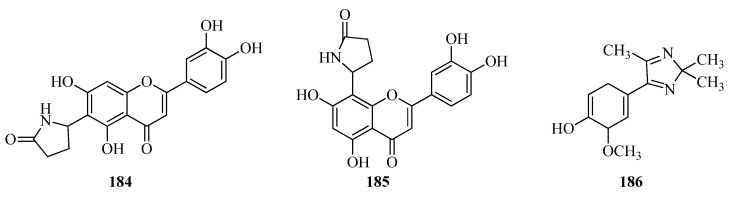
Chemical structures of alkaloid molecules.

Alkaloids in plants of the genus *Dracocephalum* have been studied to a much lesser extent compared to other groups of secondary metabolites and, according to available data, are either present in trace amounts or absent altogether. Members of this genus do not typically accumulate significant amounts of nitrogen-containing bases, which distinguishes them from many other taxa with a pronounced alkaloid profile. The contribution of alkaloids to the biological activity of *Dracocephalum* species does not appear to be decisive, yielding to phenolic compounds and terpenoids, which are considered the main bioactive metabolite groups responsible for their pharmacological effects [15]. Nevertheless, the possibility of detecting individual alkaloid-like compounds depending on the species, growing conditions, and analytical methods used cannot be ruled out, which necessitates further targeted research in this area. In addition, rare flavonoid alkaloids—dragebefin A and B (**184**, **185**)—as well as the imidazole alkaloid dragebenin C (**186**), containing a phenolic substituent (Figure 15), were isolated from *D. heterophyllum* [32]. The existence of flavonoidal alkaloids as racemates was demonstrated by HPLC using a chiral phase. Due to a stereogenic center in the pyrrolidine-2-one ring, the flavonoidal alkaloids (**184**, **185**) are chiral, although they were isolated as racemates. The enantiomers were separated by HPLC using a chiral phase and characterized stereochemically by circular dichroism spectroscopy. The structure of the compound drahebenine (**184**) was determined by X-ray crystallography.

**Figure 16:**
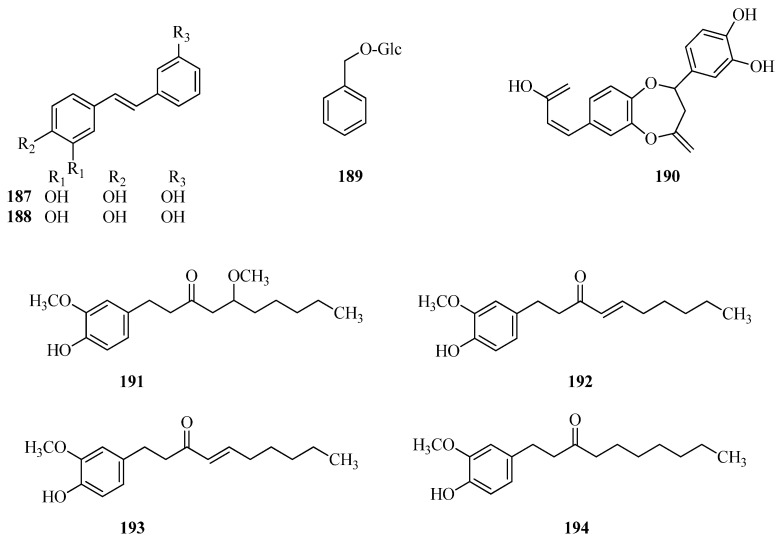
Chemical structures of molecules with different structures. Part 1.

Other phenolic compounds have been identified in some species of the genus *Dracocephalum* (Figure 16). Stilbenes in plants of the genus *Dracocephalum* have been studied only to a limited extent and, according to available data, are not among the characteristic components of their secondary metabolism, as they are present in trace amounts or are absent altogether, and their biosynthesis is weak and may occur only depending on species and growing conditions, including stress factors. In particular, the stilbenes pinocylvin (**187**) and resveratrol (**188**), as well as a benzyl alcohol glucoside (**189**), were identified in *D. ruyschiana* [97,102].

Phenylpropanoid compounds have also been detected in *D. heterophyllum* extracts—sibiricin A (**190**) and several gingerol derivatives (**191**–**194**), including 5-methoxy-6-gingerol (**191**), 6-shogaol (**192**), 6-paradol (**193**), and diacetoxy-6-gingeridol (**194**) [20].

**Figure 17:**
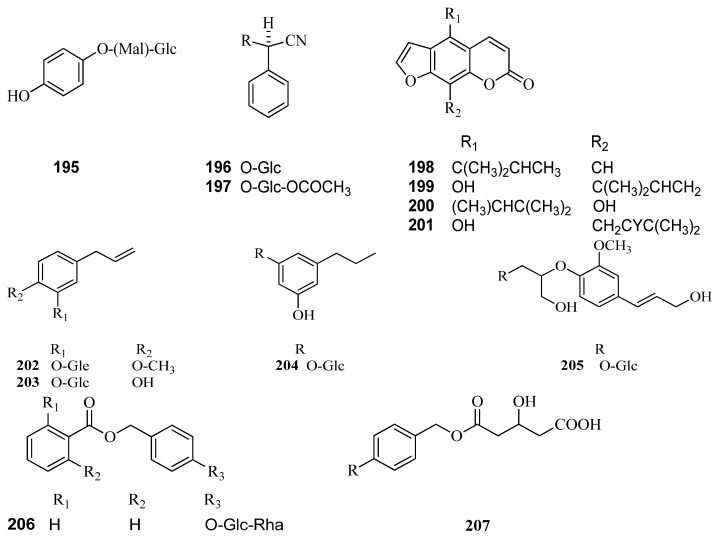
Chemical structures of molecules with different structures. Part 2.

In addition, the compound O-malonylarbutin (**195**) was identified in *D. moldavica* [117]. Cyanogenic glycosides—prunasine (**196**) and peregrinumcin A (**197**)—were isolated from *D. peregrinum* [94]. Furthermore, four compounds (**201**–**204**) belonging to the furanocoumarin group (Figure 17) were identified in *D. heterophyllum* [114].

Several phenolic glycosides were also isolated from the aerial parts of *D. moldavica*, including citrusin C (**205**), 4-allyl-1-[(β-D-glucosyl)oxy]-2-hydroxybenzene (**206**), 3,5-dihydroxyphenethyl alcohol-3-O-β-D-glucoside (**207**), and 3-hydroxy-2-[4-(3-hydroxy-1-propen-1-yl)-2-methoxyphenoxy]propyl-β-D-glucoside (**208**) [112]. Citrusin C (**205**) was also isolated from *D. ruyschiana* [102].

Complex phenolic glycosides were also isolated from *D. ruyschiana*, including [(benzoxy)methyl]phenyl-4-O-α-L-rhamnosyl-(1→6)-O-β-glucoside (**206**) and [(3-hydroxy-3-methoxyglutaryl)methyl]phenyl-4-O-β-D-glucoside (**207**) [41].

In addition, compounds belonging to other classes of secondary metabolites have been identified in the chemical composition of plants of the genus *Dracocephalum*. Steroids are present in plants of the genus *Dracocephalum* in negligible amounts. For example, phytosterols—α-sitosterol (**208**) and β-stigmasterol (**209**) [15]; these have also been found in *D. moldavica* [21].

**Figure 18:**
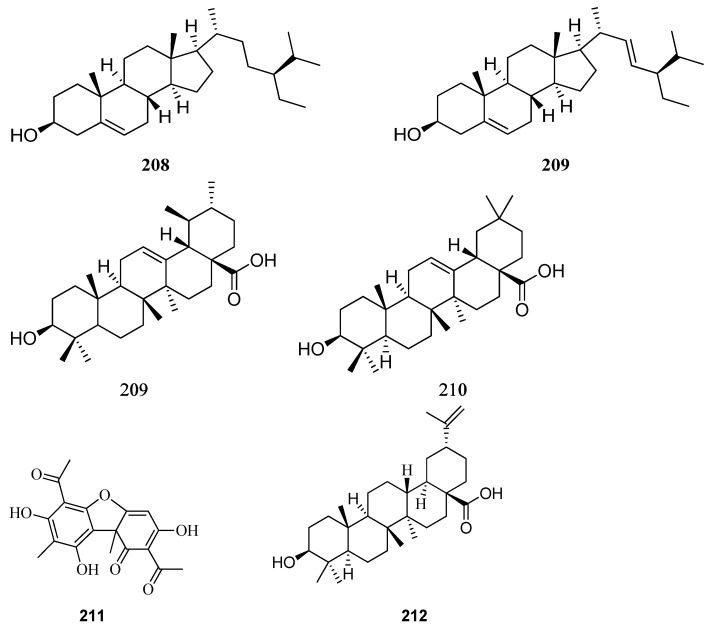
Chemical structures of diterpenoids and triterpenoids.

Diterpenoids and triterpenoids (Figure 18) in plants of the genus *Dracocephalum* are represented as structurally diverse components of secondary metabolism, with diterpenoids occurring relatively less frequently and characterized by limited distribution across species, whereas triterpenoids are more typical compounds, often accumulating in significant quantities and contributing to the biological activity of plants, including anti-inflammatory and antioxidant properties. Ursolic acid (**209**), belonging to the triterpenoid group [117], oleanolic acid (**210**) was detected in *D. moldavica*, and usnic acid (**211**) and betulinic acid (**212**) in *D. komarovii* [15].

Thus, members of the genus *Dracocephalum* are characterized by an extremely diverse chemical composition, including a wide range of secondary metabolites of various classes. In addition to phenolic acids, flavonoids, and lignans, phenylpropanoids, stilbenes, coumarins, alkaloids, cyanogenic glycosides, phenolic glycosides, and compounds from other chemical groups, such as phytosterols and triterpenoids. The presence of such a wide variety of compounds confirms the high phytochemical potential of species in the genus *Dracocephalum*. Many of the identified substances possess pronounced antioxidant, anti-inflammatory, cytotoxic, and other types of biological activity, making plants of this genus promising sources of biologically active compounds and subjects for further phytochemical and pharmacological studies.

Recent studies of the genus *Dracocephalum*, based on a combination of pharmacophylogenetic, phytochemical, and chemometric approaches, have significantly expanded our understanding of the chemical diversity and biological potential of species within this genus. It has been established that phylogenetically closely related species are characterized by similar secondary metabolite profiles and, in many cases, exhibit similar pharmacological activity, confirming the existence of a correlation between evolutionary relationship, chemical composition, and traditional medicinal use. In their analysis, the authors [6] systematized more than 900 metabolites, among which terpenoids, flavonoids, phenylpropanoids, and phenolic acids predominate.

It has been shown that phylogenetically closely related species, such as *D. moldavica*, *D. heterophyllum*, *D. nutans*, *D. integrifolium*, *D. ruyschiana*, and *D. peregrinum*, are characterized by similar secondary metabolite profiles and, in many cases, exhibit similar pharmacological activity, confirming the relationship between evolutionary kinship, chemical composition, and traditional medicinal use. Thus, species distributed in the arid and steppe regions of Kazakhstan are more often characterized by a high content of monoterpene hydrocarbons, whereas high-altitude populations of the Altai, Tien Shan, and Zhetysu Alatau are distinguished by increased accumulation of oxygen-containing terpenoids and polyphenolic compounds.

It has been established that the chemical composition and chemotypic structure of species depend significantly on climatic conditions, altitude, geographic distribution, and environmental factors. Particular attention is paid to the application of modern analytical methods, including GC–MS, metabolomic analysis, phylogenetic modeling, and network analysis of the relationships between metabolites and biological activity, which has allowed the identification of new patterns in the distribution of biologically active compounds and promising directions for the search for natural pharmacologically significant substances. The authors also emphasize that the wide geographic and ecological range of *Dracocephalum* species makes this genus a convenient model for studying the influence of environmental factors on the accumulation of secondary metabolites and the formation of chemotypic diversity.

## 5. Biological Activities

Plants of the genus *Dracocephalum* have long been used in the traditional medicine of various peoples across Eurasia. Due to their rich content of phenolic compounds, flavonoids, EOs, and other biologically active substances, members of this genus exhibit a wide range of pharmacological effects, including antioxidant, anti-inflammatory, hepatoprotective, cardioprotective, antimicrobial, and antitussive activity. The traditional use of many *Dracocephalum* species has served as the basis for their further pharmacological and phytochemical study.

One of the well-known species is *D. heterophyllum*, recognized as a traditional medicinal plant in Tibetan medicine. In Tibetan medical practice, this species is known as “Ao-Ga” or “Ji-Mei-Qing-Bao” and is used to treat various conditions, including jaundice, liver disease, cough, lymphangitis, oral ulcers, and dental disorders. In addition, in the traditional medicine of the Uyghur people, *D. heterophyllum* is used to treat certain cardiovascular and respiratory diseases [6,122,123].

Another species—*D. integrifolium* is widespread in Central Asia. In Uyghur herbal medicine, the plant is known as “Marzan Jusi” and is used as a remedy for coughs and bronchial asthma [6].

*D. peregrinum* is found in Russia, Mongolia, and northern China. In the traditional medicine of the Kazakh population of Xinjiang, this species is known as “Tekanbasdjelanbas,” and the entire plant is typically used to treat colds and various liver disorders [6,124].

The most widely studied and used species in folk medicine is *D. moldavica*. In the northern regions of Iran, this plant is known as “Badarshu,” where it is used as both a food and a medicinal plant, for example, added to yogurt or used in the form of medicinal preparations. In traditional medicine, *D. moldavica* is used for stomach and liver ailments, headaches, and nasal congestion [125]. In Eastern Europe, Siberia, Mongolia, and China, the plant is used to reduce fever, detoxify the body, relieve pain, stop bleeding, and treat coughs and bronchial asthma [6]. Additionally, in China, *D. moldavica* is a component of several pharmaceutical preparations, including Qinggan Qiwei powder, Niuhuang Shisanwei pills, and Ligan Hewei pills, used for liver diseases and other pathological conditions [126].

Recent studies show that *D. nutans* contains a significant amount of phenolic compounds, including phenolic acids and flavonoids, which account for its antioxidant activity. Extracts of the plant are capable of effectively neutralizing free radicals and exhibiting pronounced antioxidant properties. Additionally, the presence of biologically active compounds with anti-inflammatory, antimicrobial, and potentially hepatoprotective effects has been established [95].

It should be noted that phytochemical and pharmacological studies of Kazakhstani *Dracocephalum* species, including *D. nutans*, remain relatively limited compared to the more extensively studied species *D. moldavica*. However, the presence of a rich polyphenolic complex and the identified biological effects indicate the significant potential of these plants as sources of biologically active compounds. This makes the species of the genus *Dracocephalum* growing in Kazakhstan promising subjects for further phytochemical and pharmacological studies.

### 5.1. Hepatoprotective Effect

Experimental studies confirm the hepatoprotective potential of certain species of the genus *Dracocephalum*. For example, an extract of *D. heterophyllum* was shown to reduce the levels of liver enzymes, including alanine aminotransferase (ALT) and aspartate aminotransferase (AST), in the blood plasma of mice with experimental autoimmune uveitis at a dose of 20 mg/kg. The extract also decreased the concentrations of pro-inflammatory cytokines, including interferon-γ (IFN-γ) and tumor necrosis factor-α (TNF-α). These results indicate the anti-inflammatory and potentially hepatoprotective properties of this species [99].

In recent years, particular attention has been paid to studying the hepatoprotective properties of *D. moldavica*. The main biologically active components of the plant are considered to be total flavonoids, which possess antioxidant and anti-inflammatory properties. Experimental studies in models of metabolically associated fatty liver disease (MAFLD) have shown that the flavonoid fractions of *D. moldavica* are capable of reducing systemic inflammation, improving lipid metabolism parameters, and reducing the extent of liver damage [127]. It is hypothesized that the hepatoprotective effect of these compounds is associated not only with their antioxidant and anti-inflammatory properties but also with their ability to modulate the gut microbiota and maintain the integrity of the intestinal barrier. In particular, flavonoids from *D. moldavica* were shown to increase the diversity and abundance of beneficial gut microorganisms. They also reduced the translocation of lipopolysaccharides into the systemic circulation and suppressed activation of the TLR4/MyD88/NF-κB pathway in the liver. As a result, the severity of inflammatory processes and liver damage was reduced.

Thus, recent studies indicate that members of the genus *Dracocephalum* possess significant hepatoprotective potential. The biological activity of these plants is primarily due to the presence of phenolic compounds and flavonoids, which possess antioxidant, anti-inflammatory, and metabolic-regulating properties. This makes species of the genus *Dracocephalum* promising sources of natural compounds for the development of herbal preparations aimed at the prevention and treatment of liver diseases.

### 5.2. Anti-Inflammatory Activity

Anti-inflammatory drugs constitute the second-largest class of medications after anti-infective drugs. Consequently, the study of the anti-inflammatory effects of natural products has emerged as a new area of research and development for new anti-inflammatory drugs. The anti-inflammatory effects of *Dracocephalum* have also been extensively studied, including the anti-inflammatory mechanism underlying its traditional use for treating typhoid fever, the common cold, cardiovascular diseases, gastritis, sore throat, rheumatism, heart disease, and scabies [128,129].

Toshmatov et al. isolated new monoterpene glycosides—komarovin B and komarovin C—from *D. komarovii* and found that they inhibit LPS-induced NO production in macrophages, reducing inflammation at concentrations of 1, 10, 50, and 100 μM [41]. An anti-inflammatory experiment using RAW 264.7 cells showed that dracocephalumoid A, uncinaton, trichotomone F, and karyopterizoid C from *D. moldavica* can inhibit LPS-induced TNF-α, IL-1β, or NO production, exerting a significant inhibitory effect, with IC_50_ values ranging from 1.12 to 5.84 μM [129].

A study was conducted on the anti-inflammatory and gastroprotective activity of “Rozmatin” at doses of 10 mg/kg and 100 mg/kg. A dose-dependent anti-inflammatory effect was identified, with suppression of the exudative phase of inflammation, as well as a dose-dependent gastroprotective effect of “Rozmatin,” significantly exceeding the similar effect of the reference drug omeprazole. It was previously established that “Rozmatin” has a tonic effect on the cardiovascular and nervous systems [41].

Excessive production of interleukin-6 (IL-6) is linked to cytokine storm in patients with COVID-19. Blocking IL-6 has also been suggested as a therapeutic strategy for inflammatory conditions such as sepsis, a severe systemic inflammatory syndrome with high mortality. In this study, the anti-inflammatory and antiseptic effects of an ethanol extract of *D. moldavica* (DMEE), as well as its mechanisms of action in lipopolysaccharide (LPS)-induced inflammation in RAW 264.7 macrophages and in mouse models of sepsis, were investigated. It was found that DMEE reduced the release of inflammatory mediators, including nitric oxide (NO) and prostaglandin E2 (PGE2), and decreased the mRNA and protein expression of iNOS and COX-2. It also lowered the production of pro-inflammatory cytokines, mainly IL-6 and IL-1β, by inhibiting phosphorylation of JNK, ERK, and NF-κB p65. In addition, DMEE improved survival and reduced plasma IL-6 levels in mice with LPS-induced septic shock. Overall, DMEE showed anti-inflammatory and anti-septic effects by suppressing the ERK/JNK/NF-κB signaling pathways and IL-6 production [130].

### 5.3. Antimicrobial Activity

Microbial infections remain one of the leading causes of morbidity worldwide, which has heightened interest in the search for natural compounds with antimicrobial properties. Plants of the genus *Dracocephalum* have attracted significant attention from researchers due to their content of EOs, phenolic compounds, and flavonoids, which possess pronounced antibacterial and antifungal activity.

One of the most studied species is *D. moldavica*. The EO of this plant is characterized by a high content of monoterpene compounds, such as geranyl acetate, geraniol, and nerol, which play an important role in its antimicrobial activity [22]. Experimental studies have shown that the EO of *D. moldavica* effectively inhibits the growth of a number of pathogenic microorganisms, including *S. aureus*, *E. coli*, *S. typhimurium*, and *L. monocytogenes* [17].

Further studies have confirmed the pronounced antibacterial and antifungal activity of the EO of this species. It has been shown that the minimum inhibitory concentrations are approximately 0.07 mg/mL for bacteria (*B. cereus*, *B. subtilis*, *K. pneumoniae*, *S. aureus*, *M. luteus*, and *S. marcescens*) and approximately 0.08 mg/mL for fungi (*A. niger*, *R. stolonifer*, *F. oxysporum*, and *M. hiemalis*). Bioautographic analysis and subsequent identification of the active components by GC–MS showed that the main antimicrobial compounds are geraniol, nerol, geranyl acetate, geranial, neral, and neryl acetate. The results obtained indicate the potential for using *D. moldavica* EO to control pathogenic microorganisms in the food and pharmaceutical industries [74,131].

In recent years, new applications of this plant have been explored. For example, an environmentally friendly method for synthesizing silver nanoparticles (AgNPs) using an aqueous extract of *D. moldavica* seeds has been developed. The resulting nanoparticles showed strong antibacterial activity against a wide range of microorganisms, including *E. coli*, *P. aeruginosa*, *S. aureus*, *S. marcescens*, *S. epidermidis*, and *B. subtilis*. This green synthesis approach is considered a promising alternative to conventional chemical and physical methods [132].

Comparative studies of the EOs of *D. moldavica* and *H. officinalis* showed that the antimicrobial activity of *D. moldavica* is significantly higher for most of the microorganisms studied. At a concentration of 5%, the EO of *D. moldavica* produced bacterial growth inhibition zones ranging from approximately 18 to 33 mm. For some strains of *S. aureus*, the inhibition zone exceeded 40 mm. These values were significantly higher than those observed for *H. officinalis*. Even at a lower concentration (0.5%), *D. moldavica* EO retained high antimicrobial activity [133].

In addition to EOs, plant extracts also show antimicrobial activity. For example, the ethyl acetate fraction of the ethanol extract of *D. moldavica* effectively inhibited the growth of clinical isolates of *S. aureus*. The extract was shown to disrupt bacterial cell membrane integrity, suppress biofilm formation, and alter microbial energy metabolism. Proteomic analysis also revealed changes in the expression of proteins involved in metabolic and cellular processes, suggesting a complex mechanism of antimicrobial action [134].

Antimicrobial activity is also characteristic of other members of the genus. For example, the EOs of *D. integrifolium* exhibit inhibitory activity against a number of bacteria and yeasts, including *B. subtilis*, *P. aeruginosa*, *E. coli*, *S. cerevisiae*, and *C. albicans*, at minimum inhibitory concentrations in the range of 5–40 μL/mL [16].

Thus, numerous studies show that species of the genus *Dracocephalum* have strong antimicrobial activity against a wide range of bacteria and fungi. This activity is mainly associated with EO components, especially monoterpenes, as well as phenolic compounds and flavonoids. Given their broad activity and multiple mechanisms of action, *Dracocephalum* species represent a promising source of natural antimicrobial agents for the development of new pharmaceuticals and food biopreservatives.

### 5.4. Effect on Metabolic Disorders and Intestinal Inflammation

Plants of the genus *Dracocephalum* are traditionally used to treat metabolic disorders, including diabetes, hyperlipidemia, and cardiovascular diseases. In ethnomedicine, special attention is given to *D. moldavica*, whose bioactive compounds show antioxidant, anti-inflammatory, and metabolic regulatory properties.

Modern experimental studies support these traditional uses. For example, *D. moldavica* extract was shown to improve inflammatory processes associated with hypertension and intestinal dysbiosis. In a rat model of renovascular hypertension (2K1C), treatment with the extract reduced blood pressure and intestinal inflammation [135].

These effects are thought to be related to modulation of the gut microbiota. In particular, an increase in *Lactobacillus* and a decrease in opportunistic pathogens such as *Papillibacter*, *Desulfovibrio*, and *Anaerobiospirillum* were observed. These changes were accompanied by higher levels of short-chain fatty acids, which are important for intestinal metabolic and immune homeostasis.

In addition, the extract helped restore intestinal barrier integrity, reduced oxidative stress markers and pro-inflammatory factors, and suppressed the expression of key inflammatory signaling molecules, including TLR4 and NF-κB [136].

Overall, these findings suggest that *D. moldavica* has significant potential to regulate inflammatory and metabolic processes through modulation of the gut microbiota and inflammatory signaling pathways [137]. This supports the potential of *Dracocephalum* species as a source of natural compounds for the prevention and treatment of metabolic and inflammatory diseases.

### 5.5. Antitumor and Cytotoxic Activity

In recent years, considerable attention has been paid to the antitumor potential of natural compounds. Many plant metabolites can inhibit tumor cell proliferation, induce apoptosis, and modulate immune responses, making them promising candidates for anticancer drug development. In this context, species of the genus *Dracocephalum* are also regarded as a source of bioactive compounds with cytotoxic and antiproliferative properties.

Experimental studies show that different *Dracocephalum* species exhibit cytotoxic activity against various tumor cell lines. The activity depends on the plant species, extract type, and cell model used. Among them, *D. moldavica* is one of the most studied species, and several phenolic compounds with antitumor effects have been isolated from it.

A key compound is the flavonoid tilianin, a major component of the *D. moldavica* flavonoid complex. Tilianin inhibits the proliferation of human pharyngeal squamous cell carcinoma cells (FaDu), induces apoptosis, and reduces colony formation. Mechanistic studies show that it regulates the TLR4/p38/JNK/NF-κB signaling pathways, leading to activation of apoptosis. This is accompanied by decreased expression of anti-apoptotic proteins (Bcl-2, Bcl-xL) and increased levels of pro-apoptotic factors (Bad, Bax), as well as activation of caspase-3 and PARP. In addition, tilianin may modulate the tumor immune microenvironment by stimulating pro-inflammatory cytokine release and promoting dendritic cell maturation, potentially enhancing antitumor immunity [136].

Other phenolic compounds from *D. moldavica* also show cytotoxic activity. Flavones and flavonols such as diosmetin, cirsimaritin, xanthomicrol, quercetin, and kaempferol induce apoptosis in multiple myeloma (KMS-12-PE) and acute myeloid leukemia (Molm-13) cells. Diosmetin shows strong activity against myeloma cells, while cirsimaritin and xanthomicrol inhibit proliferation of acute myeloid leukemia cells and act as FLT3 tyrosine kinase inhibitors [119]. Additional antitumor effects have been reported for other compounds. The lignan dracomolfin E from *D. moldavica* inhibits the proliferation of NCI-H292 lung cancer cells, while its ethanol extract shows cytotoxic activity against DLD-1 colorectal adenocarcinoma cells.

Other species of the genus *Dracocephalum* are also of interest. For example, diosmetin from *D. peregrinum* reduces tumor formation in U251 glioma cells and induces apoptosis in vivo. In addition, flavonoid glycosides from *D. imberbe* show selective cytotoxic activity against breast (MCF-7), esophageal (OE33), and gastric (ACC201) adenocarcinoma cell lines [111].

Overall, the available data indicate that species of the genus *Dracocephalum* possess significant antitumor potential. Phenolic compounds, especially flavonoids, lignans, and caffeic acid derivatives, play a key role in these effects by inhibiting tumor cell proliferation, inducing apoptosis, and modulating immune responses. These findings support further investigation of *Dracocephalum* species as a source of novel natural antitumor agents.

### 5.6. Neuroprotective and Neuromodulatory Activity

Plants of the genus *Dracocephalum* are considered a promising source of neuroprotective compounds due to their high content of phenolic metabolites, mainly flavonoids and phenolic acids. These compounds show antioxidant, anti-inflammatory, and anti-apoptotic activities, which are important for protecting neurons in neurodegenerative and cerebrovascular diseases.

Among them, *D. moldavica* is one of the most studied species. Flavonoid-rich extracts of this plant demonstrate strong neuroprotective effects, especially in ischemia–reperfusion brain injury. In animal models, total flavonoids of *D. moldavica* reduced oxidative stress and neuronal apoptosis. This was accompanied by an increased GSH/GSSG ratio, higher activity of antioxidant enzymes, and decreased levels of oxidative stress markers such as malondialdehyde (MDA), protein carbonyls, and 8-hydroxy-2′-deoxyguanosine (8-OHdG). These changes were associated with reduced neuronal loss and improved neurological function [138].

Neuroprotective effects have also been confirmed in cell models. A flavonoid-rich extract of *D. moldavica* protected astrocytes from hydrogen peroxide (H_2_O_2_)-induced oxidative damage, indicating stabilization of antioxidant defense systems and improved cell viability [139]. In addition, ethanol extracts of the plant improved memory impairment induced by scopolamine and showed antidepressant-like effects in experimental models [140]. Aqueous extracts also produced sedative effects, reducing locomotor activity and relaxing skeletal muscles. These effects are likely related to flavonoid glycosides, including apigenin and diosmetin derivatives, which have neuromodulatory activity.

Recent studies also support the potential of *D. moldavica* in neurodegenerative diseases. In a rat model of sporadic Alzheimer’s disease induced by intracerebroventricular streptozotocin (ICV-STZ), a hydroalcoholic extract significantly improved cognitive function. The extract was standardized for phenolics, flavonoids, rosmarinic acid, and quercetin. Metabolomic analysis showed normalization of metabolites involved in disease progression, including adenine, inosine, glycerophosphoglycerol, prostaglandins, and sphingomyelin [141].

Further evidence comes from studies on vascular dementia. Bioinformatic analysis indicated that the total flavonoids of *D. moldavica* may regulate microRNAs involved in disease mechanisms. Key pathways included CaMKII, MAPK, MAPT, PI3K, and KDR, which are associated with oxidative stress, inflammation, and apoptosis. Major active compounds include kaempferol, apigenin, luteolin, and quercetin, which modulate microRNAs such as miR-3184-3p and miR-6875-5p [98].

Overall, current evidence suggests that *D. moldavica* has strong neuroprotective and neuromodulatory potential. These effects are mainly related to antioxidant activity, inhibition of neuronal apoptosis, regulation of stress-related signaling pathways, and modulation of microRNA expression. These findings support further investigation of *Dracocephalum* species as potential sources of natural compounds for the prevention and treatment of neurodegenerative and vascular brain disorders.

### 5.7. Antioxidant Activity

One of the most important pharmacological properties of plants of the genus *Dracocephalum* is their strong antioxidant activity, which is mainly related to their high content of phenolic compounds. During normal metabolism, reactive oxygen species (ROS) and other free radicals are continuously formed and play a role in cell signaling. However, their levels must be tightly controlled. An imbalance between ROS production and antioxidant defense leads to oxidative stress, which damages lipids, proteins, and nucleic acids.

Polyphenolic compounds from *Dracocephalum* species can neutralize free radicals by donating electrons or hydrogen atoms and by chelating metal ions involved in ROS formation. This reduces oxidative damage and makes these plants a promising natural source of antioxidants.

The antioxidant activity of *Dracocephalum* species has been widely studied using in vitro assays such as DPPH, ABTS, and FRAP. These methods evaluate radical scavenging and reducing capacity. Additional studies have also shown activity against superoxide, hydroxyl, and nitric oxide radicals, hydrogen peroxide, and lipid peroxidation processes [107].

In general, the strongest antioxidant activity is observed in extracts obtained with polar solvents such as methanol, ethanol, their aqueous mixtures, and ethyl acetate.

The antioxidant properties of *D. moldavica* have been studied most extensively. Leaves, flowers, and aerial parts are most often analyzed, mainly using methanol or ethanol extracts. These extracts show strong radical-scavenging and reducing activity, which strongly correlates with total phenolic and flavonoid content [107].

Chromatographic studies show that phenolic acids, especially rosmarinic and caffeic acids, are the main antioxidant components. Methanolic and hydroalcoholic extracts effectively neutralize DPPH and ABTS radicals, inhibit lipid peroxidation, and show iron-chelating activity, which further reduces ROS formation [107].

Cellular studies confirm these effects. A methanol extract of *D. moldavica* reduced bleomycin-induced DNA damage in human dermal fibroblasts, as shown by comet assay and micronucleus tests [142].

Different fractions of the extract, especially the ethyl acetate fraction rich in phenolics and flavonoids, also reduced H_2_O_2_-induced oxidative stress in cardiomyocytes. This was associated with decreased ROS levels, reduced lipid peroxidation, and regulation of apoptosis-related proteins such as Bax, Bcl-2, and caspase-3 [139]. Similar protective effects were observed in neuroglial cells, where flavonoid fractions inhibited mitochondria-dependent apoptosis and reduced oxidative damage [143].

Antioxidant activity has also been reported in other *Dracocephalum* species. For example, in *D. heterophyllum*, fractions containing rosmarinic acid, acteoside, and verbascoside showed the highest activity [120]. Other species also demonstrate strong radical-scavenging and reducing properties.

Biotechnological studies of *D. moldavica* cell cultures showed that suspension cultures accumulate high levels of rosmarinic acid and may even exhibit stronger antioxidant activity than plants grown in natural conditions [20].

The EO of *D. moldavica* also shows antioxidant activity in DPPH, ABTS, and β-carotene-linoleic acid assays, suggesting potential use as a natural food preservative [83].

Overall, the available data indicate that *Dracocephalum* species are a rich source of natural antioxidants. Their activity is mainly due to the combined effects of phenolic acids such as rosmarinic and caffeic acids, as well as flavonoids [107]. Therefore, these plants are considered promising raw materials for pharmaceuticals, functional foods, and natural antioxidant products.

### 5.8. Cardiotonic Activity

*D. moldavica* has traditionally been used in folk medicine in some regions of Iran as a cardiotonic agent. Experimental studies have shown that its total extract significantly reduces the number of ventricular tachycardia episodes, the number of ventricular extrasystoles, and the duration of ventricular tachycardia during ischemia–reperfusion. It also reduces infarct size compared with control groups. These results demonstrate a clear cardioprotective effect of the total extract in isolated rat heart models of ischemia–reperfusion injury [144].

Further studies investigated the cardioprotective effects of the total flavonoid extract of *D. moldavica* (TFDM) in vivo. The results showed that TFDM reduces myocardial apoptosis induced by ischemia–reperfusion injury. This effect is mediated mainly through activation of the PI3K/Akt/GSK-3β and ERK1/2 signaling pathways [145].

To improve its low solubility and bioavailability, TFDM was incorporated into solid lipid nanoparticles (TFDM-SLNs). The optimized nanoparticles had an average size of about 105 nm, a uniform distribution, and good stability due to a negative zeta potential. In a rat model of myocardial ischemia–reperfusion injury, both TFDM and TFDM-SLNs showed cardioprotective effects. However, TFDM-SLNs demonstrated significantly stronger protection, as shown by reduced infarct size, improved histology, and lower levels of cardiac enzymes and inflammatory markers. These results suggest that TFDM-SLNs may be an effective oral delivery system for improving the therapeutic potential of TFDM [110].

Additional in vivo studies confirmed that TFDM pretreatment reduces ischemia–reperfusion–induced myocardial injury. In rats subjected to ischemia followed by reperfusion, TFDM reduced LDH release, CK-MB, and MDA levels, increased SOD activity, and decreased infarct size, apoptosis, and mitochondrial damage. It also increased phosphorylation of PI3K, Akt, GSK-3β, and ERK1/2, while reducing pro-apoptotic proteins (Bax, cleaved caspase-3, caspase-7, and caspase-9) and increasing Bcl-2 expression. These cardioprotective effects were blocked by PI3K and ERK1/2 inhibitors, confirming the involvement of these signaling pathways [146].

In addition to cardioprotection, TFDM also shows neuroprotective effects. Similar to *D. moldavica*, it reduces the production of pro-inflammatory cytokines such as IL-8 and TNF-α [147].

Heart failure is a complex condition resulting from structural and functional cardiac impairment. TFDM has been reported to have multiple beneficial effects, including cardioprotection, anti-inflammatory activity, and reduction in oxidative stress, blood pressure, and blood glucose, as well as anti-atherosclerotic effects. However, its role in heart failure was not well understood previously. Using network pharmacology and experimental validation in a mouse model of heart failure with preserved ejection fraction (HFpEF), researchers identified multiple active compounds and molecular targets of TFDM. The results suggest that TFDM may protect the heart by reducing inflammation, apoptosis, and fibrosis, indicating its potential as a therapeutic candidate for HFpEF [148].

Network pharmacology studies combined with experimental validation also suggest that flavonoids from *D. moldavica* may protect against ischemia–reperfusion injury in the brain, such as that occurring after stroke. These compounds act on multiple targets involved in inflammation, oxidative stress, apoptosis, and neuroprotection. Overall, the findings indicate that *D. moldavica* flavonoids may exert protective effects on brain tissue through antioxidant, anti-inflammatory, and anti-apoptotic mechanisms, supporting their potential for further investigation in stroke therapy [110].

### 5.9. Other Pharmacological Effects

The EO of *D. moldavica* showed strong fumigant toxicity against adult *Sitophilus zeamais* and *Tribolium castaneum*, with LC_50_ values of 2.65 and 0.88 mg/L, respectively. It also exhibited contact toxicity against these insects, with LC_50_ values of 22.10 and 18.28 μg/adult. These results suggest that *D. moldavica* EO may be developed as a natural fumigant for controlling stored-product insect pests [90,149].

The aerial parts of *D. moldavica* are traditionally used in Central Asia and Europe for cardiovascular and skin disorders. In one study, seed extracts obtained by supercritical fluid extraction (SC-oil), ethanol (EE), and water (AE) were tested for photoprotective effects against UVB-induced photoaging in human dermal fibroblasts (HDF) and keratinocytes (HaCaT). All extracts increased type I procollagen synthesis and reduced matrix metalloproteinase-1 (MMP-1) and elastase-1 activity and expression. SC oil and AE showed the strongest effects in specific cell models. Overall, the extracts improved extracellular matrix stability and showed anti-photoaging potential due to bioactive compounds such as polyphenols, phytosterols, and omega-3 fatty acids [150].

Dried leaves of *D. moldavica* were also used as a functional food ingredient in wheat bread at concentrations of 1–5 g/100 g flour. The addition increased water absorption and total phenolic content, leading to higher antioxidant activity. However, it reduced bread volume and increased crumb hardness. Sensory evaluation showed acceptable quality up to 3 g/100 g flour, suggesting potential use as a functional food additive [151].

In traditional Mexican medicine, *D. moldavica* is used as a sedative and for nervous disorders. Experimental studies in mice showed that aqueous extracts increased pentobarbital-induced sleep time, reduced locomotor activity, and impaired motor coordination, indicating a sedative effect. However, no significant anxiolytic or antidepressant effects were observed. Toxicity testing showed an LD_50_ of 470 mg/kg, indicating moderate toxicity and general CNS depressant activity [152].

The EO of *D. moldavica* also showed analgesic properties in animal models. Its intraperitoneal LD_50_ in mice was 600 mg/kg. At doses of 5–20 mg/kg, it significantly reduced pain responses in the formalin and acetic acid-induced writhing tests, although it did not affect thermal pain sensitivity in the hot plate test. These results support the traditional use of the plant for pain relief [153].

## 6. Molecular Docking of Key Phytochemicals from the *Dracocephalum* Genus: A Review

Molecular docking is currently one of the key tools for the initial screening of the biological activity of small molecules, enabling the prediction of their affinity and spatial orientation within the active sites of target proteins. This approach is particularly relevant for the study of natural plant-derived compounds, which are characterized by a broad spectrum of biological activity and, as a rule, a more favorable safety profile compared to synthetic analogs.

In recent years, rosmarinic acid (**57**) has been intensively studied using molecular docking methods as a promising multi-target ligand of natural origin. In [154], docking was performed for the inhibition of integrin-linked kinase (ILK), the results of which demonstrated high affinity of the ligand for the enzyme’s active site. Similarly, docking of rosmarinic acid as a potential inhibitor of the TRPC1 transmembrane channel showed significant binding to the model structure (KD = 1.27 μM) [155]. Significant attention is paid to its antiviral potential: a number of studies have shown that rosmarinic acid is capable of effectively interacting with SARS-CoV-2 proteins, including the main protease (Mpro) [156] and RNA-dependent RNA polymerase (RdRp) [157], forming stable complexes with the active sites of these enzymes. Furthermore, a study [158] demonstrated its multi-target action through binding to ACE2, TMPRSS2, PLpro, and 3CLpro. In the context of neurodegenerative diseases, the affinity of rosmarinic acid for acetylcholinesterase (AChE) and β-secretase (BACE1) has been established, indicating its potential efficacy in Alzheimer’s disease [159].

Apigenin is also being actively studied using molecular docking methods as a promising natural flavonoid with significant multi-target pharmacological potential. In [160], its interaction with some receptors involved in neurodegenerative processes, including GABA, NMDA, dopamine, and serotonin receptors, was demonstrated, indicating its ability to modulate neurotransmission and neuroinflammation. In a study [161], using molecular docking and molecular dynamics methods, it was established that apigenin and its derivatives exhibit a pronounced affinity for oncogenic proteins of the human papillomavirus (HPV) and DNA polymerase θ, reaching binding energies of up to −8.8 kcal/mol. The multi-target nature of the interaction was confirmed in study [162], where apigenin demonstrated high affinity for 24 protein targets associated with inflammation, carcinogenesis, and oxidative stress, including p38 MAPK (up to −8.21 kcal/mol). Additionally, its involvement in the regulation of signaling pathways associated with breast cancer stem cells (TP53, PI3K/AKT) [163] has been demonstrated, as well as its ability to inhibit α-glucosidase [164] and interact with *Plasmodium falciparum* proteins [165]. Taken together, these data indicate the high potential of apigenin as a multi-target pharmacological agent.

Luteolin, a flavonoid structurally similar to apigenin, also demonstrates significant pharmacological potential. In study [166], a comprehensive analysis of its mechanism of action in non-small cell lung cancer (NSCLC) was conducted, including molecular docking, which demonstrated high ligand affinity for key signaling pathway proteins (TP53, EGFR, AKT1, TNF, and CASP3), indicating its involvement in the regulation of apoptosis and tumor cell proliferation. In a study [167], docking of luteolin and its derivatives to the estrogen receptor α (ER-α) was performed, revealing high binding energies (−7.2–−8.0 kcal/mol) and the formation of stable hydrophobic and π–π interactions in the protein’s active site. Additional data indicate its cardioprotective potential [168], as well as its ability to inhibit bacterial enzymes, including DNA gyrase and β-lactamases [169]. Several studies have also demonstrated luteolin’s involvement in the regulation of signaling pathways, such as JAK/STAT [170], which further confirms its multi-target action.

Diosmetin (**109**) is another promising natural flavonoid that is being actively investigated using molecular docking methods. In study [171], a comprehensive in silico analysis of the interaction between diosmetin and the CDKN2 protein, associated with prostate cancer, was conducted, where the compound demonstrated high docking scores (Gold score ≈ 58.7) and complex stability under molecular dynamics. Additionally, its effective binding to cytochrome P450 enzymes (CYP17A1 and CYP19A1) was demonstrated, where the binding energy reaches −11 kcal/mol [172]. Studies of enzymatic activity have established an interaction between diosmetin and xanthine oxidase (XOD), accompanied by inhibition of the enzyme [173]. In the context of neurodegenerative diseases, its derivatives have been developed as inhibitors of acetyl- and butyrylcholinesterase, a finding confirmed by molecular docking results [174]. The multi-target nature of diosmetin’s action has been further confirmed in network pharmacology studies [175].

Of significant interest are the monoterpene components of EOs, in particular geranial (E-citral) (**10**) and nerol (Z-citral), which are part of citral. These compounds are widely distributed in plants of the genera *Cymbopogon*, *Litsea*, and *Melissa* and possess pronounced anti-inflammatory, antimicrobial, and analgesic activity. In [176], it was shown that citral is capable of interacting with the TRPV1 channel, forming a stable complex, which is associated with its analgesic effect. Further studies demonstrate that molecular docking allows for the identification of key amino acid residues involved in the interaction of terpenoid compounds with proteins of inflammation and cellular signaling [177]. This confirms the promise of geranial and nerol as biologically active multi-target compounds.

Limonene (**21**), one of the most common monoterpenes, is also being actively studied using molecular docking methods. In study [178], its interaction with the FTO protein was demonstrated, indicating a possible role in the regulation of metabolic processes. In study [179], limonene demonstrated the ability to bind to virulence proteins of *C. albicans*, forming stable hydrophobic interactions. Additionally, its affinity for the PDE5A enzyme has been established [180], and it has been shown that its derivatives exhibit increased activity against viral proteins, including HSV-1 [181]. These data indicate a broad spectrum of biological targets for limonene.

Finally, α- and β-pinene (**18**, **1**), γ-terpinene, and β-caryophyllene are important components of EOs with proven biological potential. In [182], their interaction with proteins regulating apoptosis (p53, Bax, NF-κB) suggests potential antitumor activity. Studies of antimicrobial activity [183] have established that α- and β-pinene are capable of binding to *S. aureus* proteins, enhancing the efficacy of antibacterial therapy.

Thus, the results of modern docking studies convincingly demonstrate that natural plant-derived compounds possess significant multi-target potential, are capable of forming stable complexes with various biological targets, and are of considerable interest for the development of new pharmacological agents.

## 7. Conclusions

Thus, the species of the genus *Dracocephalum* found in Kazakhstan represent a promising source of biologically active compounds with significant pharmacological potential. Phytochemical studies have shown that they are characterized by a rich and diverse content of secondary metabolites, among which polyphenolic compounds (flavonoids and phenolic acids) and terpenoids, including EO components, predominate.

Key bioactive compounds include derivatives of apigenin, luteolin, acacetin, and diosmetin, as well as phenolic acids, primarily rosmarinic, caffeic, chlorogenic, and ferulic acids. Mono- and sesquiterpenes of EOs (citral, geraniol, 1,8-cineole, limonene, β-caryophyllene) also play an important role, forming various chemotypes depending on the species and growing conditions.

The combination of these compounds accounts for the broad spectrum of biological activities observed in *Dracocephalum* species growing in Kazakhstan, including antioxidant, anti-inflammatory, antimicrobial, gastroprotective, hypolipidemic, and cytoprotective activities. Some species also exhibit sedative, cardioprotective, and immunomodulatory properties, which is consistent with their traditional use in folk medicine.

Currently, 22 species of the genus *Dracocephalum* have been recorded in Kazakhstan. However, only a limited number of these species have been investigated with respect to their phytochemical composition and biological activities, whereas many others remain poorly studied or unexplored. This highlights the need for further comprehensive phytochemical and pharmacological investigations of this species.

Despite the accumulated data, the extent of research on these species remains uneven. The most studied are *D. moldavica*, *D. peregrinum*, and *D. ruyschiana*, whereas many endemic and rare species require in-depth phytochemical and pharmacological analysis. Promising areas for further research include the standardization of raw materials taking into account chemotypic variability, expanding the range of biological assays, and conducting preclinical and clinical studies.

Future studies on *Dracocephalum* species occurring in Kazakhstan should focus on the application of advanced metabolomic and chemometric approaches, including LC–MS/MS and GC–MS analyses combined with multivariate statistical methods for chemotype differentiation and chemotaxonomic evaluation. Additional investigations are required to clarify the influence of ecological and geographical factors on secondary metabolite biosynthesis, as well as to develop biotechnological and agronomic strategies for the standardized production of biologically active compounds. Furthermore, despite promising experimental pharmacological data, further toxicological, preclinical, and clinical studies are necessary to validate the safety, efficacy, and therapeutic potential of *Dracocephalum* species for pharmaceutical and medical applications.

Overall, *Dracocephalum* species are of significant interest as potential sources of natural compounds for the development of phytopharmaceuticals, functional foods, and preventive medicine products.

## Figures and Tables

**Table 1:** The distinctive features of previously published reviews and this review, which focuses on the species of the genus *Dracocephalum* growing in Kazakhstan.

The Main Focus of the Review	The *Dracocephalum* Species Under Consideration	Data by Region in Kazakhstan	Phytochemical Analysis	Biological Activity	Difference from This Review	Ref.
General phytochemical and pharmacological review of the *Dracocephalum* genus	China, Central Asia, and Europe	Limited	Flavonoids, phenolic acids, EOs	Antioxidant, anti-inflammatory, antimicrobial	Species growing in Kazakhstan have not been classified	[15]
EOs and chemotypes of species in the genus	Primarily Iranian and Asian species	Practically none	EOs and terpenoid composition	Antimicrobial and antioxidant activity	No analysis of Kazakhstani flora	[18]
Ethnopharmacology and traditional uses	Tibetan, Chinese, and Iranian species	Specific mentions	Polyphenols and traditional medicines	Anti-inflammatory and cardioprotective activity	Regional characteristics of species growing in Kazakhstan have not been addressed	[19]
Recent findings on polyphenols in the *Dracocephalum* genus	Different species of the genus	Limited	More than 300 phenolic compounds	Antioxidant activity	No comprehensive analysis of species growing in Kazakhstan	[20]
A comprehensive review of *Dracocephalum* species in the flora of Kazakhstan	22 species growing in Kazakhstan	Analysis of data from across Kazakhstan	EOs, phenolic acids, flavonoids, lignans, chemotypes	Antioxidant, antimicrobial, anti-inflammatory, gastroprotective, and other	Data on species growing in Kazakhstan, their chemical composition, and biological activity have been systematically compiled for the first time	This review

**Table 2:** Botanical description of 22 plants of the genus *Dracocephalum* found in Kazakhstan.

Species	Morphological Characteristics						
	Habitat	Stem	Leaves	Inflorescence	Calyx	Corolla	Ref.
*Dracocephalum bipinnatum* Bunge	Perennial	Erect, sparsely branched, 30–70 cm	Bipinnate, narrow segments	Dense terminal whorls, 6–12 flowers	Tubular-campanulate, pubescent, 6–9 mm	Blue–violet, 18–25 mm	[23]
*Dracocephalum discolor* Bunge	Perennial	Erect, slightly branched, 5–15 cm, hairy	Oblong-ovate to elliptical, slightly hairy	4–10 flowers/whorl	Tubular, 5–7 mm	Purple, 8–12 mm	[24,25]
*Dracocephalum diversifolium*	Perennial	Erect, branched above, 25–60 cm	Ovate-lanceolate to broadly ovate	Dense terminal whorls, 6–12 flowers	Tubular-campanulate, 6–9 mm	Blue-violet, 15–25 mm	[26,27,28]
*D. heterophyllum*	Perennial	Erect, 20–50 cm	Highly variable, serrate	Dense whorls, 5–10 flowers	Tubular-campanulate, 5–8 mm	Blue-violet, 15–22 mm	[29,30,31,32,33,34]
*Dracocephalum imberbe* Bunge	Perennial	Erect/ascending, 10–35 cm	Rounded-cordate to reniform	Several flowers/whorl	Elongated, 15–18 mm	Blue, 8–12 mm	[24,35,36,37]
*D. integrifolium* Bunge, (synonym). *Dracocephalum goloskokovii*	Perennial	Erect, 10–25 cm	Entire, elliptical to ovate	3–8 flowers/whorl	Tubular, 5–7 mm	Blue–purple, 8–12 mm	[16,24,38]
*Dracocephalum karataviense*	Perennial subshrub	Branched, 15–60 cm	Broadly lanceolate, glandular	3–7 flowers/whorl	Two-lipped, 8–12 mm	Bluish-purple, 18–25 mm	[39,40]
*Dracocephalum komarovii*	Perennial	Erect, 25–60 cm	Lanceolate, toothed	Dense whorls, 6–12 flowers	Tubular-campanulate, 6–8 mm	Blue-violet, 15–25 mm	[41,42,43]
*D. moldavica*	Annual	Erect, 30–80 cm	Oblong-ovate, coarsely toothed	Dense whorls, 6–15 flowers	Tubular-campanulate, 6–9 mm	Blue-lilac, 20–30 mm	[44,45,46,47,48]
*Dracocephalum nodulosum*	Perennial	Erect, 10–30 cm, hairy	Ovoid, serrate	Small clustered whorls	~10 mm	Purple-blue, ~15 mm	[49,50]
*D. nutans*	Perennial	16–55 cm	Lanceolate to elliptical	3–5 flowers/whorl	~10 mm	Purple-blue, ~20 mm	[51,52,53,54]
*Dracocephalum oblongifolium*	Perennial	Ascending, branched, 10–20 cm	Broadly ovate, serrate	Small terminal clusters	Hairy, 12–13 mm	Dark blue, 25 mm	[26,28,55,56,57]
*Dracocephalum origanoides*	Annual	Erect, 2–8 cm	Ovate, entire/slightly toothed	3–8 flowers/whorl	Tubular, 4–6 mm	Blue-purple, 8–12 mm	[58]
*Dracocephalum paulsenii* Briq	Perennial	Erect, 10–20 cm	Elliptical to ovate	4–8 flowers/whorl	Tubular, 4–6 mm	Blue-purple, 8–12 mm	[59]
*Dracocephalum pavlovii*	Perennial	Erect, 20–50 cm	Oval to lanceolate	Dense whorls, 5–10 flowers	Tubular-campanulate	Blue-violet, 12–22 mm	[36]
*Dracocephalum peregrinum*	Perennial	Erect, 20–60 cm	Oval-lanceolate, toothed	4–10 flowers/whorl	Tubular-campanulate, 5–7 mm	Pale violet, 10–18 mm	[28,43,49]
*Dracocephalum ruyschiana*	Perennial	Erect, 25–70 cm	Oblong-lanceolate	Dense whorls, 6–12 flowers	Tubular-campanulate, 6–9 mm	Blue-violet, 15–25 mm	[60,61,62,63,64]
*Dracocephalum spinulosum*	Perennial	Erect, 20–50 cm	Linear-lanceolate	Dense whorls, 4–10 flowers	Tubular-campanulate, 5–7 mm	Blue-violet, 12–20 mm	[65]
*Dracocephalum stamineum*	Perennial	Erect	Lanceolate, serrate	Dense whorls, 4–10 flowers	Tubular-campanulate, 5–7 mm	Blue-violet, 12–20 mm	[66]
*D. thymiflorum*	Annual	Erect, 10–60 cm	Ovate to oblong-lanceolate	6–12 flowers/whorl	Cup-shaped, 6–8 mm	Purple-blue, 7–10 mm	[67,68,69]

**Table 3:** Chemical composition of essential oils of *Dracocephalum*.

Species	Location	Number of Identified Compounds	Main Compounds	Ref.
D. diversifolium Rupr.	Uzbekistan	29	β-pinene (1)—10.6%, sabinene (2)—20.7%, m-cymene (3)—6.2%	[70]
*D. heterophyllum* Benth.	Western Himalayas	40	Citronellol (**4**)—74.2%	[71]
	India (cultivated species)	-	Citronellyl acetate (**5**)—14%	[72]
	China	83	1.8-cineole (**6**)—14.9%, trans-nerolidol (**7**)—7.10%, germacrene-D (**8**)—4.84%	[30]
*D. integrifolium* Bunge	China, alpine pasture	24	Sabinene (**2**)—14%, 1.8-cineole (**6**)—52.56%	[38]
	China, riverbank area	21	Sabinene (**2**)—7.35%, 1.8-cineole (**6**)—76.1%	[38]
	China, valley	21	Sabinene (**2**)—10.1%, 1.8-cineole (**6**)—65.6%	[38]
*D. moldavica*	Egypt	-	Linalool (**9**)—16.8–37.5%, geranial (**10**)—11.6–24.2%	[73]
	Egypt	-	Neral (**11**)—11.99%, geraniol (**12**)—14.96%, geranial (**10**)—23.67%, geranyl acetate (**13**)—24.93%	[74]
	Iran	-	Neral (**11**)—11.99%, geraniol (**12**)—14.96%, geranial (**10**)—23.67%, geranyl acetate (**13**)—24.93%	[75]
	Iran	34	Geranial (**10**)—33.94%, neral (**11**)—22.4%, geranyl acetate (**13**)—19.27%	[76]
	Iran	-	Geranial (**10**)—31.14%, neral (**11**)—31.5%, geranyl acetate (**13**)—4.3%	[14]
	Egypt	-	Geranial (**10**)—19.13–35.19%, neral (**11**)—17.82–18.83%, geranyl acetate (**13**)—18.97–30.36%	[77]
	Turkey	-	Geranial (**10**)—23.7–27.6%, neral (**11**)—17.7–20.2%, geranyl acetate (**13**)—36.5–43.3%	[78]
	Egypt	42	Geranial (**10**)—19.37–20.42%, neral (**11**)—17.85–18.35%, geranyl acetate (**13**)—27.02–28.81%	[48]
	Egypt	-	Geranial (**10**)—22.57–24.56%, neral (**11**)—19.93–20.56%, geranyl acetate (**13**)—28.85–29.60%	[79]
	Iran	-	Neral (**11**)—16.25%, geraniol (**12**)—24.31%, geranyl acetate (**13**)—36.62%	[80]
	Ukraine	-	Neral (**11**)—10.25–43.49%, geraniol (**12**)—3.35–28.14%, geranial (**10**)—11.52–42.45%, geranyl acetate (**13**)—0.13–14.65%	[81]
	Iran	-	Neral (**11**)—21.21%, geraniol (**12**)—24.31%, geranyl acetate (**13**)—36.62%	[17]
	Iran	-	Neral (**11**)—13.38–21.26%, geraniol (**12**)—16.86–22.05%, geranyl acetate (**13**)—22.51–24.72%	[82]
	Iran	22	Neral (**11**)—21.9–28.57%, geranial (**10**)—29.08–39.44%, geranyl acetate (**13**)—24.68–34.8%	[83]
	Serbia	88	Geranial (**10**)—29,6%, geranyl acetate (**13**)—27.2%, neral (**11**)—19.4%	[84]
	Belarus	17	Citral (**14**)—87.21%	[85]
	Iran	70	Geranial (**10**)—25.5%, geranyl acetate (**13**)—15.2%	[86]
	Mongolia	55	Limonene-10-al (**15**)—75%	[87]
	Serbia	34	Linalool acetate (**16**)—10.7%, geranial (**10**)—16.8%, geranyl acetate (**13**)—53.2%	[22]
	Poland	47	Geranial (**10**)—33.94%, neral (**11**)—32.00%, geraniol (**12**)—9.29%	[88]
	Iran	13	Neral (**11**)—32.1%, geranial (**10**)—21.6%, geranyl acetate (**13**)—19.9%, geraniol (**12**)—17.6%	[89]
	Iran	-	Geranyl acetate (**13**)—27.81–28.81%, geranial (**10**)—19.37- 20.36%; neral (**11**)—17.85–18.26%	[49]
	China	38	1.8-Cineol (**6**)—31.25, 4-terpineol (**17**)—22.82%	[90]
	Ukraine	19	Geranyl acetate (**13**)—41.08%, geranial (**10**)—25.5%, neral (**11**)—18.85%	[21]
*D. nutans*	Kazakhstan	22	1.8-cineole (**6**)—34%, α-pinene (**18**)—6.7%, β-pinene (**1**)—5.2%, β-myrcene (**19**)—5.3%	[91]
*D. oblongifolium* Regel	Tajikistan	82	Isopinocamphone (**20**)—38.45%, limonene (**21**)—6.11%	[92]
*D. paulsenii* Briq.	Afghanistan	-	α-pinene (**18**)—24.5%, S-(-)-cis-verbenol (**22**)—14.5%, germacrone (**23**)—14.1%, caryophyllene oxide (**24**)—13.6%	[93]
*D. peregrinum*	Kazakhstan	49	1.8-cineol (**6**)—18.5%, α -pinene (**18**)—8.4%, limonene (**21**)—5.8%, caryophyllene oxide (**24**)—5.5%	[94]
	Mongolia	35	Linalool (**9**)—17.9%	[87]
*D. ruyschiana*	Mongolia	98	Thymol (**25**)—34.0%, carvacrol (**26**)—6.1%	[87]
	Kazakhstan	46	α-pinene (**18**)—3.8%	[95]
*D. thymiflorum*	Kazakhstan	52	β-caryophyllene oxide (**24**)—12.2%, spathulenol (**27**)—9.4%	[96]

## Data Availability

No new data were created or analyzed in this study.

## References

[1] Uritu C.M., Mihai C.T., Stanciu G.-D., Dodi G., Alexa-Stratulat T., Luca A., Leon-Constantin M.-M., Stefanescu R., Bild V., Melnic S. (2018). Medicinal plants of the family Lamiaceae in pain therapy: A review. Pain Res. Manag..

[2] Zvezdina E.V., Dayronas Z.V., Bochkareva I.I., Zilfikarov I.N., Babaeva E.Y., Ferubko E.V., Guseynova Z.A., Serebryanaya F.K., Kaibova S.R., Ibragimov T.A. (2020). Representatives of the Family Lamiaceae Lindl. as Sources of Medicinal Plant Raw Materials for Neurotropic Drugs (Review). Pharm. Pharmacol..

[3] Levaya Y., Atazhanova G., Gabe V., Badekova K. (2025). A Review of Botany, Phytochemistry, and Biological Activities of Eight *Salvia* Species Widespread in Kazakhstan. Molecules.

[4] Hazrati S., Habibzadeh F., Davatgar-Iranizad A., Ebadi M.-T. (2026). Changes in essential oil content and composition of *Dracocephalum multicaule* at different phenological stages: Determination of balsamic time. Biochem. Syst. Ecol..

[5] Engler A., Prantl K. (1887). Die Natürlichen Pflanzenfamilien.

[6] Liu H., Feng X., Zhao Y., Lv G., Zhang C., Aruhan, Damba T.A., Zhang N., Hao D., Li M. (2024). Pharmacophylogenetic Relationships of Genus *Dracocephalum* and Related Genera Based on Multifaceted Analysis. Front. Pharmacol..

[7] Ferreira E.d.O., Fernandes M.Y., Lima N.M., Neves K.R., Carmo M.R., Lima F.A., Fonteles A.A., Menezes A.P., Andrade G.M. (2016). Neuroinflammatory response to experimental stroke is inhibited by eriodictyol. Behav. Brain Res..

[8] Zhang C.J., Li W., Li H.Y., Song Y., Zhao X.W. (2009). In Vivo and In Vitro Antiviral Activity of Five Tibetan Medicinal Plant Extracts Against Herpes Simplex Virus Type 2 Infection. Pharm. Biol..

[9] Ballabh B., Chaurasia O.P. (2007). Traditional Medicinal Plants of Cold Desert Ladakh Used in Treatment of Cold, Cough and Fever. J. Ethnopharmacol..

[10] Selenge E., Murata T., Tanaka S., Sasaki K., Batkhuu J., Yoshizaki F. (2014). Monoterpene Glycosides, Phenylpropanoids, and Acacetin Glycosides from *Dracocephalum foetidum*. Phytochemistry.

[11] Heydari P., Yavari M., Adibi P., Asghari G., Ghanadian S.M., Dida G.O., Khamesipour F. (2019). Medicinal Properties and Active Constituents of *Dracocephalum kotschyi* and Its Significance in Iran: A Systematic Review. Evid. Based Complement. Altern. Med..

[12] Borghei S.F., Azizi A., Pourhosseini S.H., Rahimi-Rizi M. (2024). Characterization of dragonhead (*Dracocephalum moldavica* L.) landraces: Genetic, chemotypic, and agro-morphologic perspectives. J. Appl. Res. Med. Aromat. Plants.

[13] Dastmalchi K., Dorman H.J.D., Laakso I., Hiltunen R. (2007). Chemical Composition and Antioxidative Activity of Moldavian Balm (*Dracocephalum moldavica* L.) Extracts. LWT Food Sci. Technol..

[14] Fattahi M., Nazeri V., Torras-Claveria L., Sefidkon F., Cusido R.M., Zamani Z., Palazon J. (2013). Identification and Quantification of Leaf Surface Flavonoids in *Dracocephalum kotschyi*. Food Chem..

[15] Zeng Q., Jin H., Fu J., Qin J., Hu H., Yan L., Chen M., Zhang W. (2010). Chemical Constituents of Plants from the Genus *Dracocephalum*. Chem. Biodivers..

[16] Zhou S., Wei C., Zhang C., Han C., Kuchkarova N., Shao H. (2019). Chemical Composition, Phytotoxic, Antimicrobial and Insecticidal Activity of the Essential Oils of *Dracocephalum integrifolium*. Toxins.

[17] Ehsani A., Alizadeh O., Hashemi M., Afshari A., Aminzare M. (2017). Phytochemical, Antioxidant and Antibacterial Properties of *Melissa officinalis* and *Dracocephalum moldavica* Essential Oils. Vet. Res. Forum.

[18] Rezaei-Chiyaneh E., Javanmard A., Seyyedi S.M., Battaglia M.L., Moghaddam S.S., Etesami H. (2021). Improvement of dragonhead (*Dracocephalum moldavica* L.) yield quality through a coupled intercropping system and vermicompost application along with maintenance of soil microbial activity. Land Degrad. Dev..

[19] Wang J., Sun J., Wang M., Cui H., Zhou W., Li G. (2022). Chemical Constituents from *Dracocephalum moldavica* and Their Chemotaxonomic Significance. Biochem. Syst. Ecol..

[20] Weremczuk-Jeżyna I., Grzegorczyk-Karolak I. (2025). Phenolic Compounds in *Dracocephalum* Genus and Their Biological Activities. Molecules.

[21] Barchuk O., Pryshlyak A., Shanaida M. (2021). Chemical Composition and Sedative Activities of Essential Oils of *Dracocephalum moldavica* and *Ocimum americanum*. Pharmacologyonline.

[22] Aćimović M., Stanković J., Cvetković M., Kiprovski B., Sikora V., Zeremski T., Brdar-Jokanović M., Popović V., Dolijanović Ž., Nestorović Živković J. (2022). Chemical Composition, Antioxidant, and Antimicrobial Activity of *Dracocephalum moldavica* Essential Oil and Hydrolate. Plants.

[23] Dai L., Zhao Y., Wang M., Liu J., Li X., Yang P. (2008). A New Ferulic Acid Ester and Other Constituents from *Dracocephalum peregrinum*. Arch. Pharmacal Res..

[24] Lazkov G.A., Veselova P.V. (2022). About Two Species of the Genus *Dracocephalum*. Turczaninowia.

[25] Malik R.A., Gupta R., Kumari S., Gupta B.K. (2017). New Chromosome Reports in Lamiaceae of Kashmir (Northwest Himalaya), India. Protoplasma.

[26] Dobrea D.I., Călina D., Bădulescu L., Chira L., Toma F. (2017). The Influence of Nutrition Space on Herb and Seed Yields of *Dracocephalum moldavica* L. under ARDS Secuieni Pedoclimatic Conditions. Sci. Pap. Ser. A Agron..

[27] Abdullaeva N.S., Ortiqova L.S., Isabekova M.A. (2021). Phytocenotic Description of *Dracocephalum* Species in the Flora of Uzbekistan. Turk. J. Comput. Math. Educ..

[28] Abdullaeva N.S., Khodzhimatov O.K., Azimova D.E. (2019). The Genus *Dracocephalum* L. in the Phytogeographical Regions of Uzbekistan. Am. J. Plant Sci..

[29] Zhang C., Liu Y., Wang X., Li H., Zhao J. (2008). Chemical Composition and Biological Activity of Essential Oil of *Dracocephalum heterophyllum*. Nat. Prod. Res..

[30] Numonov S.R., Qureshi M.N., Aisa H.A. (2015). HPLC Quantification of Flavonoids from *Dracocephalum heterophyllum*. Int. J. Anal. Chem..

[31] Numonov S.R., Usmanova S.K., Aisa H.A. (2013). Chemical Composition of *Dracocephalum heterophyllum*. Chem. Nat. Compd..

[32] Fu G., Li X., Wang Y., Zhang H., Chen L., Zhao Y. (2022). Characterization of the Complete Chloroplast Genome of the Dragonhead Herb, *Dracocephalum heterophyllum* (Lamiaceae), and Comparative Analyses with Related Species. Diversity.

[33] Makhkamov T.K., Akbarov F.I., Rakhmatova D.K. (2021). Distribution of *Dracocephalum diversifolium*. Rupr. Acad. Res. Educ. Sci..

[34] Denisova G.R., Cheremushkina V.A., Astashenkov A.Y. (2022). Changes in the Ontogenetic Spectrum and Density of the Cenopopulation of *Dracocephalum imberbe* Bunge (Lamiaceae) in the Republic of Tuva (Russia) over 17 Years. Rastit. Mir Aziat. Ross..

[35] *Dracocephalum imberbe* Bunge. Plants of the World Online. Royal Botanic Gardens, Kew. https://powo.science.kew.org/results?q=Dracocephalum%20imberbe.

[36] Chen Y.-P., Turdimatovich T.O., Nuraliev M.S., Lazarević P., Drew B.T., Xiang C.-L. (2022). Phylogeny and biogeography of the northern temperate genus *Dracocephalum* s.l. (Lamiaceae). Cladistics.

[37] Rostovtseva T.S. (1978). Cytomixis in *Dracocephalum imberbe* Bunge (Lamiaceae). Tsitologiya I Genet..

[38] Zhang R.-Q., Ma X.-L., Chen Y.-P., Xiang C.-L. (2024). Complete chloroplast genome sequences of *Dracocephalum argunense* and *D. integrifolium* (Lamiaceae: Nepetinae). J. Asia-Pac. Biodivers..

[39] Abdullaeva N.S. (2016). Distribution of *Dracocephalum* L. Genus Species in Uzbekistan’s Flora. Uchenyi XXI Veka.

[40] Khasanov D.U., Zhalolov I.Z., Dolimov K.K., Aripova S.F. (2024). Chemical Constituents of *Dracocephalum paulsenii*. Chem. Nat. Compd..

[41] Toshmatov Z.O., Karimov O.K., Ismailov A.I., Rakhimov B.S., Yusupov S.A. (2019). New Monoterpene Glucosides from *Dracocephalum komarovi* and Their Anti-Inflammatory Activity. Phytochem. Lett..

[42] Uchiyama N., Takahashi K., Matsumoto K., Yamamoto H., Kato S. (2003). New Icetexane and 20-Norabietane Diterpenes with Trypanocidal Activity from *Dracocephalum komarovi*. J. Nat. Prod..

[43] Uchiyama N., Takahashi K., Matsumoto K., Yamamoto H., Kato S. (2004). A Trypanocidal Diterpene with Novel Skeleton from *Dracocephalum komarovi*. Tetrahedron Lett..

[44] Yang L.N., Wang H., Li J., Zhao Q., Chen W. (2014). Phenolic Compounds from *Dracocephalum moldavica* L.. Biochem. Syst. Ecol..

[45] Gao T., Wu L., Ma Y., Zhai W., Zhang L., Cong W., Cai Z., Cui C., Li L. (2025). Pickering emulsion loaded with total flavonoids from *Dracocephalum moldavica* L. potentially promotes angiogenesis in the ischemic penumbra after cerebral ischemia–reperfusion. Front. Bioeng. Biotechnol..

[46] Wu P., Yan X.S., Zhou L.L., Liu X.L., Huo D.S., Song W., Jia J.X. (2019). Involvement of apoptosis in the protective effects of *Dracocephalum moldavica* in a cerebral ischemia–reperfusion rat model. J. Toxicol. Environ. Health Part A.

[47] Ose A., Andersone-Ozola U., Ievinsh G. (2021). Substrate-Dependent Effect of Vermicompost on Yield and Physiological Indices of Container-Grown *Dracocephalum moldavica* Plants. Agriculture.

[48] Said-Al Ahl A.H., Sabra A.S., El Gendy A.N.G., Aziz E.E., Tkachenko K.G. (2015). Changes in Content and Chemical Composition of *Dracocephalum moldavica* L. Essential Oil at Different Harvest Dates. J. Med. Plants Stud..

[49] Telepova M.N., Budantzev A.L., Shavarda A.L. (1992). Comparative Study of Terpene Secretion by Leaf Glandular Elements in Different Species of the Genus *Dracocephalum* L.. Bull. Société Bot. Fr. Lett. Bot..

[50] Denisova G.R., Azimshoeva M.A. (2010). Morphogenesis of *Dracocephalum nodulosum* Rupr. Vestnik Voronezhskogo Gosudarstvennogo Universiteta. Seriya: Geografiya. Geoekologiya.

[51] Azimshoeva M.A., Denisova G.R. (2011). Morphology of Seedlings and Juvenile Plants of Some Species of the Genus *Dracocephalum* L.. Dokl. Akad. Nauk Resp. Tadzhikistan.

[52] Sagdullayevna A.N., Nozima U. (2021). Analysis of Herbarium Samples of *Dracocephalum* L. Distributed in Uzbekistan. Int. J. Orange Technol..

[53] Sabiyeva A., Ishmuratova M.Y., Atazhanova G.A., Smagulov M.K., Kurmantaeva G.K., Ashirbekova B.B., Taiken A.A. (2023). Anatomical Study of *Dracocephalum ruyschiana* L. and *Dracocephalum nutans* L.. Res. J. Pharm. Technol..

[54] Gordeeva N.I. (2019). Features of Reproduction and Inheritance of Female and Hermaphrodite Phenotypes of the Gynodioecious Species *Dracocephalum nutans* (Lamiaceae). Rastit. Mir Aziat. Ross..

[55] Khaksar S., Kiarostami K., Alinaghi S. (2022). The Effects of Methanol Extracts of *Hyssopus officinalis* on a Model of Induced Glioblastoma Multiforme (GBM) in Rats. J. Mol. Neurosci..

[56] Shavarda A., Budantsev A. (2013). Analysis of *Dracocephalum oblongifolium* (Lamiaceae) Essential Oil Using Comprehensive Two-Dimensional Chromatography. J. Essent. Oil Res..

[57] Moridi Farimani M., Mirzania F., Sonboli A., Moghaddam F.M. (2017). Chemical Composition and Antibacterial Activity of *Dracocephalum kotschyi* Essential Oil Obtained by Microwave-Assisted Extraction and Hydrodistillation. Int. J. Food Prop..

[58] Beshko N.Y., Khojimatov O.K., Bussmann R.W. (2023). *Dracocephalum* Species in Uzbekistan. Ethnobiology of Uzbekistan: Ethnomedicinal Knowledge of Mountain Communities.

[59] Abdullaeva N.S., Khozhimatov O.K., Kubakova K.K. (2020). Synopsis of species of the genus *Dracocephalum* L. (Lamiaceae) growing in Uzbekistan. Sci. Herit..

[60] Dastmalchi-Razgonova M., Okhlopkova Z., Golokhvast K. (2022). Research of *Dracocephalum palmatum* S. and *Dracocephalum ruyschiana* L. originating from Yakutia and identification of metabolites by tandem mass spectrometry. BIO Web Conf..

[61] Weremczuk-Jeżyna I., Gomulski J., Kiss A.K., Grzegorczyk-Karolak I. (2024). Effect of Ag^+^ and Cd^2+^ elicitation on polyphenol production in shoot culture of *Dracocephalum ruyschiana* L.. Molecules.

[62] Koohdar F., Sheidai M. (2021). Biosystematic study in some *Dracocephalum* species (Lamiaceae) based on morphology and anatomy in Iran. Acta Bot. Hung..

[63] Sabiyeva A., Ishmuratova M.Y., Atazhanova G.A., Smagulov M.K., Zhuravel I.A. (2022). Histochemical analysis of aerial parts of *Dracocephalum ruyschiana* L. and *Dracocephalum nutans* L. growing in Central Kazakhstan. Res. J. Pharm. Technol..

[64] Lazarević P., Stevanović V., Niketić M. (2009). On the Distribution of *Dracocephalum ruyschiana* (Lamiaceae) in the Balkan Peninsula. Phytol. Balc..

[65] Naderifar M., Sonboli A., Gholipour A. (2015). Pollen morphology of Iranian *Dracocephalum* L. (Lamiaceae) and its taxonomic significance. Bangladesh J. Plant Taxon..

[66] Beshko N.Y., Khojimatov O.K., Bussmann R.W. (2023). *Dracocephalum bipinnatum* Rupr.; *Dracocephalum diversifolium* Rupr.; *Dracocephalum formosum* Gontsch.; *Dracocephalum heterophyllum* Benth.; *Dracocephalum imberbe* Bunge; *Dracocephalum integrifolium* Bunge; *Dracocephalum komarovii* Lipsky; *Dracocephalum nodulosum* Rupr.; *Dracocephalum nutans* L.; *Dracocephalum oblongifolium* Regel; *Dracocephalum paulsenii* Briq.; *Dracocephalum stamineum* Kar. & Kir. Ethnobiology of Uzbekistan: Ethnomedicinal Knowledge of Mountain Communities.

[67] Talovskaya E.B., Cheryomushkina V.A., Denisova G.R. (2018). Morphological adaptation of *Thymus* (Lamiaceae) species in Yakutia. Contemp. Probl. Ecol..

[68] Koohdar F., Sheidai M., Attar F. (2015). Population genetic structure and genetic diversity in *Dracocephalum thymiflorum* L. (Lamiaceae) populations in Iran. Mol. Plant Breed..

[69] Abbasov N.K., Ganbarov D.S., Seyidov M.M. (2024). A New Record for the Flora of Azerbaijan: *Dracocephalum thymiflorum* L.. Bull. Sci. Pract..

[70] Melieva S.O., Bobakulov K.B., Abdullaeva N.S., Karimov A., Eshbakova K.A. (2020). Essential oil and volatile components of *Dracocephalum diversifolium*. Khim. Rastit. Syr’ya.

[71] Stappen I., Wanner J., Tabanca N., Wedge D.E., Ali A., Kaul V.K., Lal B., Jaitak V., Gochev V.K., Schmidt E. (2015). Chemical composition and biological activity of essential oils of *Dracocephalum heterophyllum* and *Hyssopus officinalis*. Nat. Prod. Commun..

[72] Singh N., Kaul V.K., Megeji N.W., Singh V., Ahuja P.S. (2008). Essential oil composition of three accessions of *Dracocephalum heterophyllum*. Nat. Prod. Res..

[73] Hussein M.S., El-Sherbeny S.E., Khalil M.Y., Naguib N.Y., Aly S.M. (2006). Growth characters and chemical constituents of *Dracocephalum moldavica* L.. Sci. Hortic..

[74] El-Baky H.H., El-Baroty G.S. (2008). Chemical and biological evaluation of essential oil of *Dracocephalum moldavica*. Int. J. Integr. Biol..

[75] Omidbaigi R., Yavari S., Hassani M.E. (2010). Introduction of autoploidy in *Dracocephalum moldavica*. J. Fruit Ornam. Plant Res..

[76] Sheykhjan M.G., Fazlara A., Hojjati M., Behbahani B.A. (2025). Active food packaging strategies using *Dracocephalum moldavica* essential oil. Appl. Food Res..

[77] Aziz E.E., Hussein M.S., Wahba H.E., Razin A.M. (2013). Essential oil constituents of *Dracocephalum moldavica* under salt stress. Middle East J. Sci. Res..

[78] Eshan K.A., Bial G., Kiarash A.P.R., Mesut U. (2014). GC/MS analysis of bioactive components of *Dracocephalum moldavica* L., treated by boric acid doses. Tarım Bilim. Araştırma Derg..

[79] Hegazy M.H., Abdel-Rahman E.A., El-Sayed M.A., Ahmed K.A., Abdel-Mogib M. (2016). Effects of zinc on Lamiaceae plants. Eur. J. Med. Plants.

[80] Golparvar A.R., Ghorbani R., Hadipanah A., Yadegari S. (2016). Chemical constituents of *Dracocephalum moldavica* and *Dracocephalum kotschyi*. Acta Agric. Slov..

[81] Kotyuk L.A., Rakhmetov D.B. (2017). Component composition of essential oil from *Dracocephalum moldavica* L. grown in the Ukrainian Polissya. Conservation of Plant Diversity.

[82] Janmohammadi M., Sufi-Mahmoudi Z., Ahadnezhad A., Yousefzadeh S., Sabaghnia N. (2014). Influence of chemical and organic fertilizer on growth, yield and essential oil of dragonhead (*Dracocephalum moldavica* L.) plant. Acta Agric. Slov..

[83] Fallah S., Rostaei M., Lorigooini Z., Sukri A.A. (2018). Chemical compositions of essential oil and antioxidant activity of *Dracocephalum moldavica* in sole crop and dragonhead–soybean intercropping system under organic manure and chemical fertilizers. Ind. Crops Prod..

[84] Aćimović M., Stanković J., Cvetković M., Todosijević M., Rat M. (2019). The chemical composition of the essential oil of *Dracocephalum moldavica* L. from Vojvodina Province (Serbia). Biol. Nyssana.

[85] Romanova M.G., Buzuk A.G. (2020). Chemical composition of essential oil of *Dracocephalum moldavica*. Vestn. Farmatsii.

[86] Fattahi A., Shakeri A., Tayarani-Najaran Z., Harbakh M. (2021). UPLC–PDA–ESI–QTOF–MS/MS and GC-MS analysis of *Dracocephalum moldavica*. Food Sci. Nutr..

[87] Özek G., Ozek G., Demirci B., Baser K.H.C. (2016). Preparative Capillary GC for Characterization of Five *Dracocephalum* Essential Oils from Mongolia, and Their Mosquito Larvicidal Activity. Nat. Prod. Commun..

[88] Dmitruk M., Weryszko-Chmielewska E., Sulborska A., Kwiecień I., Misiak L., Piotrowska K. (2019). Sites of secretion of bioactive compounds in *Dracocephalum moldavica*. Braz. J. Bot..

[89] Sonboli A., Mojarrad M.J., Gholipour A.R., Yousefzadi M., Arman M. (2008). Biological Activity and Composition of the Essential Oil of *Dracocephalum Moldavica* L. Grown in Iran. Nat. Prod. Commun..

[90] Chu S.S., Liu S.L., Liu Q.Z., Liu Z.L., Du S.S. (2011). Composition and toxicity of Chinese *Dracocephalum moldavica* (Labiatae) essential oil against two grain storage insects. J. Med. Plants Res..

[91] Atazhanova G., Sabiyeva A., Akhmetova S., Smagulov M., Medeshova A., Sarsembayeva S., Sarsembayeva A., Aldabayeva U., Kurmantaeva G. (2023). Component composition and antimicrobial activity of *Dracocephalum nutans* essential oil. Res. J. Pharm. Technol..

[92] Shvarda A.L., Budantsev A.L. (2013). Analysis of essential oil of *Dracocephalum oblongifolium*. Rastit. Resur..

[93] Jeppesen A.S., Soelberg J., Jäger A. (2012). Chemical composition of essential oils from medicinal plants. J. Essent. Oil Bear. Plants.

[94] Suleimen E.M., Myrzagalieva A.B., Ibataev Z.A., Iskakova Z.B., Samarkhanov T.N., Medeubaeva B.Z. (2017). Constituent composition and biological activity of essential oil from *Dracocephalum peregrinum*. Chem. Nat. Compd..

[95] Sabiyeva A., Atazhanova G., Zholdasbaev M., Ashirbekova B., Medeshova A., Kurmantaeva G., Turdiyeva Z., Sarsembayeva S., Ibraibekova A., Mukasheva G. (2025). Development of formulation and technology of an antimicrobial gel with essential oil of *Dracocephalum nutans* L. grown in Central Kazakhstan. Res. J. Pharm. Technol..

[96] Sheidai M., Koohdar F. (2018). Statistical evaluations of morphological and anatomical characteristics of *Dracocephalum thymiflorum*. (Lamiaceae) populations in Iran. Acta Bot. Hung..

[97] Okhlopkova Z.M., Razgonova M.P., Pikula K.S., Zakharenko A.M., Piekoszewski W., Manakov Y.A., Ercisli S., Golokhvast K.S. (2022). *Dracocephalum palmatum* S. and *Dracocephalum ruyschiana* L. originating from Yakutia: A high-resolution mass spectrometric approach for the comprehensive characterization of phenolic compounds. Appl. Sci..

[98] Liu M., Shan G., Jiang H., Zeng L., Zhao K., Li Y., Ashraf G.M., Li Z., Liu R. (2021). Identification of miRNA and their regulatory effects induced by total flavonoids from *Dracocephalum moldavica* in the treatment of vascular dementia. Front. Pharmacol..

[99] Zheng W., Wang Q., Lu X., Shi Q., Zou J., Tao Y., Wang P. (2016). Protective effects of *Dracocephalum heterophyllum* in ConA-induced acute hepatitis. Mediat. Inflamm..

[100] Jiang H., Zhang C., He W. (2018). The effects of *Dracocephalum heterophyllum* Benth flavonoids on hypertrophic cardiomyocytes induced by angiotensin II in rats. Med. Sci. Monit..

[101] Nasiri Y. (2021). Crop productivity and chemical composition of *Dracocephalum moldavica* L. essential oil under different cropping patterns and fertilization. Ind. Crops Prod..

[102] Selenge E., Murata T., Kobayashi K., Batkhuu J., Yoshizaki F. (2013). Flavone tetraglycosides and benzyl alcohol glycosides from *Dracocephalum ruyschiana*. J. Nat. Prod..

[103] Olennikov D.N., Chirikova N.K., Okhlopkova Z.M., Zulfugarov I.S. (2013). Chemical Composition and Antioxidant Activity of *Tánara Ótó* (*Dracocephalum palmatum* Stephan), a Medicinal Plant Used by the North-Yakutian Nomads. Molecules.

[104] Zamani S., Bakhshi D., Ebadi M.-T., Sahraroo A. (2025). Phytochemical profile and antioxidant activity of *Dracocephalum kotschyi* Boiss. affected by environmental conditions of cultivation regions. BMC Plant Biol..

[105] Weremczuk-Jeżyna I., Grzegorczyk-Karolak I., Frydrych B., Królicka A., Wysokińska H. (2013). Hairy roots of *Dracocephalum moldavica*: Rosmarinic acid content and antioxidant potential. Acta Physiol. Plant..

[106] Li Q., Liu Y., Han L., Liu J., Liu W., Feng F., Zhang J., Xie N. (2016). Chemical constituents and quality control of two *Dracocephalum* species based on high-performance liquid chromatographic fingerprints coupled with tandem mass spectrometry and chemometrics. J. Sep. Sci..

[107] Simea Ș., Ielciu I., Hanganu D., Niculae M., Pall E., Burtescu R.F., Olah N.-K., Cenariu M., Oniga I., Benedec D. (2023). Evaluation of the Cytotoxic, Antioxidative and Antimicrobial Effects of *Dracocephalum moldavica* L. Cultivars. Plants.

[108] Sheichenko O.P., Sheichenko V.I., Goryainov S.V., Zvezdina E.V., Kurmanova E.N., Ferubko E.V., Uyutova E.V., Potanina O.G., Khajjar F. (2021). Chemical composition and biological activity of the dry extract “Rozmatin” from the herb of Moldavian dragonhead (*Dracocephalum moldavica* L.). Chem. Plant Raw Mater..

[109] Shanaida M., Golembiovska O., Hudz N., Wieczorek P. (2018). Phenolic compounds of herbal infusions obtained from some species of the Lamiaceae family. Curr. Issues Pharm. Med. Sci..

[110] Zheng R.-F., Kader K., Liu D.-W., Su W.-L., Xu L., Jin Y.-Y., Xing Y.-G. (2024). A network pharmacology approach to decipher the mechanism of total flavonoids from *Dracocephalum moldavica* L. in the treatment of cardiovascular diseases. BMC Complement. Med. Ther..

[111] Fu P., Zhao C.C., Tang J., Shen Y.H., Xu X.K., Zhang W.D. (2009). Triterpenoids and flavonoids from the chloroform fraction of *Dracocephalum peregrinum*. Chem. Nat. Compd..

[112] Wang L., Wang S., Yang S., Guo X., Lou H., Ren D. (2012). Phenolic alkaloids from the aerial parts of *Dracocephalum heterophyllum*. Phytochemistry.

[113] Sultan A., Bahang H.A., Eshbakova A.K.A. (2008). Flavonoids from *Dracocephalum moldavica*. Chem. Nat. Compd..

[114] Fu P., Zhao C.C., Tang J., Shen Y.H., Xu X.K., Zhang W.D. (2009). New flavonoid glycosides and cyanogenic glycosides from *Dracocephalum peregrinum*. Chem. Pharm. Bull..

[115] Khushmatov S.S., Usmanov P.B., Zaripov A.A., Esimbetov A.T., Gashmatov Z.O., Hadji Akbar A., Mirkhodjaev U.Z., Babaev S.N., Radzhabova G.G. (2018). Relaxant effect of the flavonoid penduletin isolated from plants of the genus *Dracocephalum komarovii* Lipsky (Labiatae). Soil, Climate, Fertilizer and Yield: Current Problems and Perspectives.

[116] Hu X., Mola Y., Su W.-L., Wang Y., Zheng R.-F., Xing J.-G. (2023). A network pharmacology approach to decipher the total flavonoid extract of *Dracocephalum moldavica* L. in the treatment of cerebral ischemia–reperfusion injury. PLoS ONE.

[117] Kilincarslan S., Salieva K., Borkoev B., Aldayarov N., Kaya B., Nalbantsoy A., Boke Sarikahya N. (2025). Chemical constituents with cytotoxic activity from *Dracocephalum imberbe*. ChemistrySelect.

[118] Zhang H., Wang S., Liu Q., Zheng H., Liu X., Wang X., Shen T., Ren D. (2021). Dracomolphin A–E, new lignans from *Dracocephalum moldavica*. Fitoterapia.

[119] Jöhrer K., Galarza M., Kircher B., Çiçek S.S. (2022). Flavones, flavonols, lignans, and caffeic acid derivatives from *Dracocephalum moldavica* and their in vitro effects on multiple myeloma and acute myeloid leukemia. Int. J. Mol. Sci..

[120] Dang J., Wang S., Shao Y. (2018). Preparative isolation of antioxidative compounds from *Dracocephalum heterophyllum* using off-line two-dimensional reversed-phase LC/HILIC guided by on-line HPLC–DPPH assay. J. Chromatogr. B.

[121] Zhang H., Xu L., Liu X., Fan J., Wang X., Shen T., Wang S., Ren D. (2020). Dracomolphesin A–E, five 3,4-seco-phenylpropanoids with Nrf2-inducing activity from *Dracocephalum moldavica*. Chin. Chem. Lett..

[122] Lv Y., Li C., Wang Z., Wang Q., Li G., Dang J. (2022). Preparative isolation of antioxidative furanocoumarins from *Dracocephalum heterophyllum* and their potential action target. J. Sep. Sci..

[123] Jiang H., He W., Zhang C. (2018). Effect of total flavonoids of *Dracocephalum heterophyllum* on norepinephrine-induced cardiomyocyte hypertrophy in rats. Chin. J. Pharmacol. Toxicol..

[124] Yan Y.L. (2020). Chemical Constituents and Antitumor Activity of *Dracocephalum peregrinum*. Ph.D. Thesis.

[125] Morshedloo M.R., Amani Machiani M., Mohammadi A., Maggi F., Soleimani Aghdam M., Mumivand H., Javanmard A. (2020). Comparison of drying methods for the extraction of essential oil from dragonhead (*Dracocephalum moldavica* L., Lamiaceae). J. Essent. Oil Res..

[126] Xing J.H., Zhao A.G., Bao F.G., Liu Q.L. (2021). Stnuy on standardized planting technology of Mongolian medicine *Cymbidium Mongolica*. J. N. Pharm..

[127] Sun X., Ge N., Liang Q., Wang Q., Yu H., Jin M. (2025). Effect of the total flavonoids of *Dracocephalum moldavica* L. on metabolic-associated fatty liver disease in rats. Front. Pharmacol..

[128] Minaiyan M., Sadraei H., Asghari G., Khanabadi M. (2017). Anti-inflammatory effect of apigenin and hydroalcoholic extract of *Dracocephalum kotschyi* on acetic acid-induced colitis in rats. Res. Pharm. Sci..

[129] Nie L., Li R., Huang J., Wang L., Ma M., Huang C., Wu T., Yan R., Hu X. (2021). Abietane diterpenoids from *Dracocephalum moldavica* L. and their anti-inflammatory activities in vitro. Phytochemistry.

[130] Kim K.-M., Kim S.-Y., Mony T.J., Bae H.J., Han S.-D., Lee E.-S., Choi S.-H., Hong S.H., Lee S.-D., Park S.J. (2021). *Dracocephalum moldavica* Ethanol Extract Suppresses LPS-Induced Inflammatory Responses through Inhibition of the JNK/ERK/NF-κBSignaling Pathway and IL-6 Production in RAW 264.7 Macrophages and in Endotoxic-Treated Mice. Nutrients.

[131] Rezaie Keikhaie K., Jahantigh H.R., Bagheri R., Rezaie Kehkhaie A. (2018). The effects of the ethanol extract of *Dracocephalum moldavica* (Badrashbu) against strains of antibiotic-resistant *Escherichia coli* and *Klebsiella pneumoniae*. Int. J. Infect..

[132] Haghighi Pak Z., Abbaspour H., Karimi N., Fattahi A. (2016). Eco-Friendly Synthesis and Antimicrobial Activity of Silver Nanoparticles Using *Dracocephalum moldavica* Seed Extract. Appl. Sci..

[133] Avagyan A., Martirosyan G., Brindza J., Sargsyan G., Vardanian I., Harutyunyan Z., Balayan R., Harutyunyan M., Hovhannisyan M., Tadevosyan L. (2025). Antimicrobial activity of essential oils from introduced varieties of *Dracocephalum moldavica* and *Hyssopus officinalis*. Funct. Foods Sci..

[134] Yu H., Liu M., Liu Y., Qin L., Jin M., Wang Z. (2019). Antimicrobial activity and mechanism of action of *Dracocephalum moldavica* L. extracts against clinical isolates of Staphylococcus aureus. Front. Microbiol..

[135] Yu H., Chen Z., Chen H., Wang Z. (2023). Intestinal inflammation and microbiota regulation: Effects of *Dracocephalum moldavica* extract. Arch. Biol. Sci..

[136] Jiang H., Zeng L., Dong X., Guo S., Xing J., Li Z., Liu R. (2020). Tilianin extracted from *Dracocephalum moldavica* L. induces intrinsic apoptosis and regulates inflammatory microenvironment response in pharyngeal squamous carcinoma cells via TLR4 signaling pathway. Front. Pharmacol..

[137] Kurmanova E.N., Ferubko E.V., Sheychenko O.P. (2019). Evaluation of anti-inflammatory and gastroprotective properties of *Dracocephalum moldavica* extract. Vopr. Biol. Meditsinskoi I Farmatsevticheskoi Khimii.

[138] Sun Y., Liu T., Dai X., Jiang Z., Gao Z., Zhang M., Wang D., Zheng Q. (2014). Neuroprotective effect of *Dracocephalum moldavica* L. total flavonoids in transient cerebral ischemia in rats. Annu. Res. Rev. Biol..

[139] Jin M., Yu H., Jin X., Yan L., Wang J., Wang Z. (2020). *Dracocephalum moldavica* L. extracts protect H9c2 cardiomyocytes against H_2_O_2_-induced apoptosis and oxidative stress. BioMed Res. Int..

[140] Shafiee-Kandjani A., Khalili M., Malek A., Farhang S., Ranjbari Y., Khalili Y. (2023). The therapeutic effect of the extracts of *Lavandula angustifolia* and *Dracocephalum ruyschiana* besides sertraline on patients with mild to moderate depression: A double-blind controlled trial. Phytomed. Plus.

[141] Talebi M., Ayatollahi S.A., As’Habi M.A., Kobarfard F., Khoramjouy M., Boroujeni F.N., Faizi M., Ghassempour A. (2025). Investigating the neuroprotective effects of *Dracocephalum moldavica* extract and its effect on metabolomic profile of rat model of sporadic Alzheimer’s disease. Heliyon.

[142] Aprotosoaie A.C., Mihai C.T., Vochița G., Rotinberg P., Trifan A., Luca S.V., Petreus T., Gille E., Miron A. (2016). Antigenotoxic and antioxidant activities of a polyphenolic extract from European *Dracocephalum moldavica* L. *Ind*. Crops Prod..

[143] Zheng R.-F., Du Y.-W., Zeng C., Wang H.-F., Xing J.-G., Xu M. (2020). Total flavones of *Dracocephalum moldavica* L. protect astrocytes against H_2_O_2_-induced apoptosis through a mitochondria-dependent pathway. BMC Complement. Med. Ther..

[144] Najafi M., Ghasemian E., Fathiazad F., Garjani A. (2009). Effects of total extract of *Dracocephalum moldavica* on ischemia/reperfusion-induced arrhythmias and infarct size in the isolated rat heart. Iran. J. Basic Med. Sci..

[145] Tan M.-E., He C.-H., Jiang W., Zeng C., Yu N., Huang W., Gao Z.-G., Xing J.-G. (2017). Development of solid lipid nanoparticles containing total flavonoid extract from *Dracocephalum moldavica* L. and their therapeutic effect against myocardial ischemia–reperfusion injury in rats. Int. J. Nanomed..

[146] Zeng C., Jiang W., Yang X., He C., Wang W., Xing J. (2018). Pretreatment with total flavonoid extract from *Dracocephalum moldavica* L. attenuates ischemia–reperfusion-induced apoptosis. Sci. Rep..

[147] Jia J.X., Zhang Y., Wang Z.L., Yan X.S., Jin M., Huo D.S., Wang H., Yang Z.J. (2017). The inhibitory effects of *Dracocephalum moldavica* L. on rat cerebral ischemia reperfusion injury. J. Toxicol. Environ. Health Part A.

[148] Dawuti A., Sun S., Wang R., Gong D., Yuan T., Zhang L., Yang S., Xing J., Zheng R., Lu Y. (2022). Systems pharmacology-based strategy to investigate pharmacological mechanisms of total flavonoids from *Dracocephalum moldavica* on chronic heart failure. Int. J. Mol. Sci..

[149] Ding S.E., Cixi T., Charles P. (2015). Synthesis and toxicity of Chinese *Dracocephalum moldavica* (Lamiaceae) essential oil against two grain storage insects. Afr. J. Insects.

[150] Song E., Choi J., Gwon H., Hwang J. (2025). Protective effect of *Dracocephalum moldavica* L. seed extracts against ultraviolet B-induced photoaging in human skin cells. J. Nutr. Health.

[151] Dziki D., Cacak-Pietrzak G., Gawlik-Dziki U., Sułek A., Kocira S., Biernacka B. (2019). Effect of Moldavian dragonhead (*Dracocephalum moldavica* L.) leaves on the baking properties of wheat flour and quality of bread. CYTA J. Food.

[152] Martínez-Vázquez M., Estrada-Reyes R., Martínez-Laurrabaquio A., López-Rubalcava C., Heinze G. (2012). Neuropharmacological study of *Dracocephalum moldavica* L. (Lamiaceae) in mice: Sedative effect and chemical analysis of an aqueous extract. J. Ethnopharmacol..

[153] Maham M., Akbari H., Delazar A. (2013). Chemical composition and antinociceptive effect of the essential oil of *Dracocephalum moldavica* L.. Pharm. Sci..

[154] Wahab S., Alshehri S.A., Usmani S., Almoyad M.A.A., Ahmad W. (2025). Identification of rosmarinic acid as a potent inhibitor of integrin-linked kinase (ILK) with anticancer activity in breast cancer cells. S. Afr. J. Bot..

[155] Quan W., Wang Y., Chen Y.H., Shao Q., Gong Y.Z., Hu J.W., Liu W.H., Wu Z.J., Wang J., Ma S.B. (2023). Screening of rosmarinic acid from *Salvia miltiorrhiza* acting on the novel target TRPC1 based on the homology modelling–virtual screening–molecular docking–affinity assay–activity evaluation method. Pharm. Biol..

[156] Patel U., Desai K., Dabhi R.C., Maru J.J., Shrivastav P.S. (2023). Bioprospecting phytochemicals of *Rosmarinus officinalis* L. for targeting SARS-CoV-2 main protease (Mpro): A computational study. J. Mol. Model..

[157] Selvaraj J., Rekha U.V., Jh S.F., Sivabalan V., Ponnulakshmi R., Vishnupriya V., Kullappan M., Sreekandan R.N., Mohan S.K. (2021). Molecular docking analysis of SARS-CoV-2 linked RNA-dependent RNA polymerase (RdRp) with natural compounds including rosmarinic acid. Bioinformation.

[158] Kumar S., Kumar V. (2024). Rosmarinic acid as a potential multi-targeted inhibitor for SARS-CoV-2: An in silico virtual screening approach. Curr. Drug Targets.

[159] Borges R.S., Ortiz B.L.S., Pereira A.C.M., Keita H., Carvalho J.C.T. (2019). *Rosmarinus officinalis* essential oil: A review of its phytochemistry, anti-inflammatory activity, and mechanisms of action involving molecular docking. J. Ethnopharmacol..

[160] Wasim M., Zia S.R., Ahmad S. (2024). Molecular docking analysis reveals the promising role of apigenin as a neuroprotective agent. World Neurosurg..

[161] Akash S., Bayıl I., Hossain M.S., Islam M.R., Hosen M.E., Mekonnen A.B., Nafidi H.A., Bin Jardan Y.A., Bourhia M., Bin Emran T. (2023). Novel computational and drug design strategies for inhibition of human papillomavirus-associated cervical cancer and DNA polymerase theta receptor by apigenin derivatives. Sci. Rep..

[162] Peanlikhit T., Aryal U., Welsh J.S., Shroyer K.R., Rithidech K.N. (2025). Evaluation of the Inhibitory Potential of Apigenin and Related Flavonoids on Various Proteins Associated with Human Diseases Using AutoDock. Int. J. Mol. Sci..

[163] Ou M., Deng Z., Shi Y., Li J., Wang X., Zhang H., Chen Y., Liu Y. (2024). Mechanism of apigenin against breast cancer stem cells: Network pharmacology and experimental validation. Front. Pharmacol..

[164] Ren X., Liu X., Wang B., Zhang M., Wang L. (2024). Inhibitory mechanism of apigenin, quercetin, and phloretin on α-glucosidase. Food Biosci..

[165] Hermanto F., Syam A.K., Haq F.A., Rachmawan R.L. (2023). Structure-based drug design method: Molecular docking study and pharmacophore modelling of apigenin as an antimalarial. Int. J. Appl. Pharm..

[166] Zhang J., Li C., Li W., Shi Z., Liu Z., Zhou J., Tang J., Ren Z., Qiao Y., Liu D. (2024). Mechanism of luteolin against non-small-cell lung cancer: A study based on network pharmacology, molecular docking, molecular dynamics simulation, and in vitro experiments. Front. Oncol..

[167] Atwon F.J., Da’I M. (2025). Molecular docking study of luteolin and its derivatives for identifying potential ER-α inhibitors in breast cancer. J. Pharm. Sci..

[168] Aneke C., Amoah A.S., Salimon S.S. (2025). Network pharmacology and molecular docking analysis of luteolin from *Ginkgo biloba* in cardiovascular diseases. Biochem. Mosc. Suppl. Ser. A.

[169] Diyah N.W., Indriani D.A., Dessidianti R., Siswandono S. (2024). In silico analysis of luteolin derivatives as antibacterial agents targeting DNA gyrase and CTX-M-15 extended-spectrum β-lactamase of *Escherichia coli*. J. Adv. Pharm. Technol. Res..

[170] Guo F., Guo Y., Zhang D., Fu Z., Han S., Wan Y., Guan G. (2024). Luteolin inhibits the JAK/STAT pathway based on network pharmacology, molecular docking and molecular dynamics simulation. Toxicol. Appl. Pharmacol..

[171] Sheoran S., Sharma R., Kumar A., Singh P., Gupta R., Meena S. (2023). In silico analysis of diosmetin as an effective chemopreventive agent against prostate cancer: Molecular docking and simulation study. J. Biomol. Struct. Dyn..

[172] Oak C., Khalifa A.O., Isali I., Bhaskaran N., Walker E., Shukla S. (2018). Diosmetin suppresses human prostate cancer cell proliferation through the induction of apoptosis and cell cycle arrest. Int. J. Oncol..

[173] Liu Y., Chen H., Xiang H., Zhang X., Wang Y., Li J., Zhao K. (2020). Inhibition and molecular mechanism of diosmetin against xanthine oxidase by multiple spectroscopies and molecular docking. New J. Chem..

[174] Yang A., Yi X., Zhang H., Shen R., Kou X. (2024). Diosmetin derivatives as multifunctional anti-AD ligands: Design, synthesis, and biological evaluation. Chem. Biol. Drug Des..

[175] Garg M., Chaudhary S.K., Goyal A., Sarup P., Kumari S., Garg N., Vaid L., Shiveena B. (2022). Comprehensive review on therapeutic and phytochemical exploration of diosmetin: A promising moiety. Phytomed. Plus.

[176] Santos S.A.A.R., Damasceno M.B.M.V., Magalhães F.E.A., Sessle B.J., Oliveira B.A., Batista F.L.A., Vieira-Neto A.E., Campos A.R. (2022). Transient receptor potential channel involvement in antinociceptive effect of citral in orofacial acute and chronic pain models. EXCLI J..

[177] Caballero J. (2022). A new era for the design of TRPV1 antagonists and agonists with the use of structural information and molecular docking. J. Enzym. Inhib. Med. Chem..

[178] Maddah M., Hoseinian N., Pourfath M. (2023). An ensemble docking-based virtual screening according to different TRPV1 pore states toward identifying phytochemical activators. New J. Chem..

[179] Ahmed M.Z., Hameed S., Ali M., Hizbullah S., Zaheer A. (2021). In silico molecular docking analysis of limonene with the fat mass and obesity-associated protein by using AutoDock Vina. Sci. J. Inform..

[180] Ahmedi S., Pant P., Raj N., Manzoor N. (2022). Limonene inhibits virulence associated traits in *Candida albicans*: In vitro and in silico studies. Phytochem. Plus..

[181] Azzahra F., Nadapdap E.G.O., Cahyani N., Salsabila F.A., Afada M.M., Auli W.N., Saputro A.H. (2025). Molecular docking of piperine, limonene, and eugenol in *Piper nigrum* L. as anti-stroke. Pharm. Rep..

[182] Islam M.R., Islam Sovon M.S., Amena U., Rahman M., Hosen M.E., Kumer A., Bourhia M., Bin Jardan Y.A., Ibenmoussa S., Wondmie G.F. (2024). Ligand-based drug design against Herpes Simplex Virus-1 capsid protein by modification of limonene through in silico approaches. Sci. Rep..

[183] Cimen Acikgul F., Duran N., Kutlu T., Ay E., Tek E., Bayraktar S. (2024). The therapeutic potential and molecular mechanism of alpha-pinene, gamma-terpinene, and p-cymene against melanoma cells. Heliyon.

